# FOXP3+ regulatory T cells are associated with the severity and prognosis of sarcoidosis

**DOI:** 10.3389/fimmu.2023.1301991

**Published:** 2023-12-20

**Authors:** Karen C. Patterson, Wallace T. Miller, Wayne W. Hancock, Tatiana Akimova

**Affiliations:** ^1^Division of Pulmonary, Allergy, and Critical Care Medicine, Perelman School of Medicine, University of Pennsylvania, Philadelphia, PA, United States; ^2^Department of Radiology, Perelman School of Medicine, University of Pennsylvania, Philadelphia, PA, United States; ^3^Division of Transplant Immunology, Department of Pathology and Laboratory Medicine, and Biesecker Center for Pediatric Liver Diseases, Children’s Hospital of Philadelphia and Perelman School of Medicine, University of Pennsylvania, Philadelphia, PA, United States

**Keywords:** regulatory T-cells, Treg, sarcoidosis, sCD25, Ki-67, TNFRI, TNFRII, TNFα

## Abstract

**Rationale:**

Sarcoidosis is an inflammatory granulomatous disease of unknown etiology with predominant lung involvement. Organ involvement and disease severity, as well as the nature of immune alterations, vary among patients leading to a range of clinical phenotypes and outcomes. Our objective was to evaluate the association of disease course and immune responses in pulmonary sarcoidosis.

**Methods:**

In this prospective cohort study of 30 subjects, most of whom were followed for one year, we evaluated 14 inflammatory markers in plasma, 13 Treg/T cell flow cytometry markers and 8 parameters of FOXP3^+^ Treg biology, including suppressive function, epigenetic features and stability.

**Results:**

We identified a set of 13 immunological parameters that differ in sarcoidosis subjects in comparison with healthy donors. Five of those were inversely correlated with suppressive function of Tregs in sarcoidosis, and six (TNFα, TNFR I and II, sCD25, Ki-67 and number of Tregs) were particularly upregulated or increased in subjects with thoracic lymphadenopathy. Treg suppressive function was significantly lower in patients with thoracic lymphadenopathy, and in patients with higher burdens of pulmonary and systemic symptoms. A combination of five inflammatory markers, Ki-67 expression, Treg function, and lung diffusion capacity evaluated at study entry predicted need for therapy at one year follow-up in 90% of cases.

**Conclusion:**

Tregs may suppress ongoing inflammation at local and systemic levels, and TNFα, TNFR I and II, sCD25 and Ki-67 emerge as attractive biomarkers for *in vivo* sarcoid inflammatory activity.

## 1 Introduction

Sarcoidosis is a disabling and potentially life-threatening granulomatous disease of unknown etiology. Nearly any organ in the body may be affected, but the lungs are the most common site of disease. Outcomes in sarcoidosis are highly variable. Following diagnosis, many patients enter remission with a favorable prognosis. However, those with chronically active disease often require prolonged treatment, and have an increased risk of fibrosis. Understanding the mechanisms of chronically active sarcoidosis is a critical unmet need.

The pathophysiology of sarcoidosis includes an increase in pro-inflammatory cytokines and chemokines, influx and accumulation of T cells and macrophages and, ultimately, the formation of granulomas. Impairments in immunoregulatory mechanisms are thought to contribute to uncontrolled and persistent inflammation (1). Studies have shown alterations in the numbers and function of FOXP3^+^ regulatory T cells (Treg), although data across different studies are conflicting. While an increased number of circulating Tregs is a consistent finding (2–5), Treg counts have been associated with worse outcome in some (3, 6) but not all studies (4). Similarly, data on Treg suppressive function vary, with impaired (2, 3), partially impaired (6), or normal (5) function reported, and one study showing no association with clinical features (5) with another demonstrating an association of restored Treg function with remission (2).

The aim of our study was to further investigate if Tregs are important in the pathophysiology of sarcoidosis by measuring the association of Treg features with the disease course in a clinically well-characterized cohort of patients with pulmonary involvement. We also investigated the association of clinical data with Treg features and with a comprehensive panel of immune markers which have been previously reported to be altered in sarcoidosis or implicated in disease activity (**Supplementary Table S1**).

## 2 Materials and methods

### 2.1 Subjects

Eligible adult subjects seen in the in the pulmonary clinic at the Hospital of University of Pennsylvania, were serially recruited and enrolled via informed consent. Patients with biopsy-confirmed sarcoidosis were included in our analysis (**Table 1**). The study was approved by the hospital’s Institutional Review Board (#819345).

**Table 1:** Clinical features of sarcoidosis subjects.

#	Organs affected by sarcoidosis	Thoracic LAN	Years btw diagnosis and enrollment	Treatment-naïve at enrollment? Type of treatment and indication	Type of treatment and indication at 1 year of follow-up
1	Lungs (51-75%), heart, extra-thoracic LN	No	0.15	No Tx	Steroids
2	Lungs (51-75%)	*No imaging data*	0.44	No Tx	No follow-up data
3	Lungs (51-75%), heart, spleen	Yes	1.29	Steroids	IFX, refractory disease
4	Lungs (51-75%), heart, liver	No	3.64	Steroids + MTX	No Tx
5	Lungs (51-75%)	Yes	0.09	No Tx	No Tx
6	Lungs (6-25%), skin	No	1.95	Steroids	No Tx
7	Lungs (26-50%)	Yes	0.94	No Tx	No Tx
8	Lungs (51-75%), heart	Yes	10.96	Steroids + MTX	No Tx
9	Lungs (76-100%)	No	0.59	No Tx	No Tx
10	Lungs (26-50%)	Yes	0.07	No Tx, but Abatacept (CTLA4–Ig) for RA	No Tx
11	Lungs (26-50%)	Yes	0.32	No Tx	Steroids + IFX
12	Lungs (51-75%)	No	1.76	MTX 25 mg weekly	No Tx
13	Lungs (<5%)	Yes	1.75	No Tx	No Tx
14	Lungs (26-50%), spleen	Yes	3.02	No Tx	No Tx
15	Lungs (6-25%), eyes, CNS, spleen	Yes	0.07	No Tx	Steroids
16	Lungs (<5%)	Yes	0.00	No Tx	No follow-up data
17	Lungs (6-25%), spleen, skin	No	0.01	No Tx	No Tx
18	Lungs (<5%), eyes, skin, extra-thoracic LN	Yes	5.76	Acthar Gel	Steroids
19	Lungs (51-75%)	Yes	0.00	No Tx	Steroids
20	Lungs (6-25%), spleen	*No imaging data*	0.00	No Tx	No Tx
21	Lungs (6-25%)	Yes	0.00	No Tx	No follow-up data
22	Lungs (26-50%), heart	Yes	8.11	Steroids	No Tx
23	Lungs (26-50%), extra-thoracic LN	No	2.08	No Tx	No Tx
24	Lungs (51-75%)	Yes	2.51	No Tx	Steroids
25	Lungs (51-75%)	Yes	0.11	Steroids	No Tx
26	Lungs (<5%)	Yes	0.01	No Tx	No Tx
27	Lungs (51-75%)	No	0.20	No Tx	No Tx
28	Lungs (<5%), heart, liver	No	0.80	No Tx	No Tx
29	Lungs (26-50%), heart	Yes	31.6*	No Tx	No Tx
30	Lungs (51-75%), eyes, skin, extra-thoracic LN	Yes	0.53	No Tx	HD

CNS, central nervous system; RA, rheumatoid arthritis; LN, lymph node(s).

LAN = thoracic lymphadenopathy, >1 cm in short diameter on computed tomography imaging.

Tx, treatment; Steroids, systemic corticosteroids, equivalent of at least 10 mg/day of prednisone; MTX, methotrexate; IFX, Infliximab, monoclonal antibody against tumor necrosis factor-alpha; HD, hydroxychloroquine.

The radiographic extent of disease in lungs shown in brackets.

*While this patient had a remote history of sarcoidosis, for concern of recurrent disease he had undergone lung biopsy (with findings of active sarcoid inflammation) at the time of blood sample collection for this study and at time of imaging to assess for LAN.

### 2.2 Radiology review

Chest computed tomography (CT) imaging review was performed for each subject, and included assessment of central peri-bronchial nodular thickening, parenchymal nodules, fibrosis (traction bronchiectasis, architectural distortion, fibrotic bands, and/or honeycombing), and thoracic lymphadenopathy (> 1 cm in shortest diameter). Central peri-bronchial disease was limited to the main airways and first two segmental divisions, as the physiologic consequences of peri-bronchial disease affecting large and medium airways may differ from small airways disease, and nodules associated with smaller divisions can be difficult to distinguish from non-airway lesions. In addition to specific features, the extent of disease was graded (< 5%, 6-25%, 26-50%, > 50%), **Table 1**.

### 2.3 Clinical data

Clinical data were abstracted from the electronic medical record and recorded in REDCap (Research Electronic Data Capture) (7). CT images were reviewed for a range of features by a thoracic radiologist with expertise in sarcoidosis and blinded to clinical details. Pulmonary function testing was performed near the time of study entry, using established criteria (8). Chronically active disease was established by the need for systemic immunosuppression at one year follow-up. Pulmonary symptoms assessed included dyspnea, cough, wheezing, chest tightness, chest pain, sputum production, and hemoptysis. For our analyses, we included not only the presence but also the number of pulmonary symptoms, as any single symptom may be non-specific for sarcoidosis (9). While subjective, other studies also have correlated symptoms with disease status (9). Dyspnea was graded by the patient as either trivial/non-limiting or non-trivial/limiting in some capacity. Systemic symptoms including fevers, sweats, weight loss, fatigue, and joint pain or body pain (not attributable to other known causes) also were captured.

### 2.4 Donors

Healthy donor peripheral blood mononuclear cells (PBMCs) were obtained through the University of Pennsylvania Human Immunology Core. Healthy donor plasma (matched by age, gender, race and smoking history to the sarcoidosis cohort, **Supplementary Table S2**) was obtained from Innovative Research, USA and used for Luminex assay. All donors provided informed consent.

### 2.5 Treg isolation and functional studies

After Ficoll separation of blood from sarcoidosis patients, plasma was cryopreserved for Luminex assay, and CD4^+^CD25^+^ Tregs were isolated from PBMCs with a Treg isolation kit (#130-091-301, Miltenyi Biotec) and immediately used in suppression assay as described (10). Shortly, isolated Treg in serial dilutions 1/1 through 1/16 were incubated with carboxyfluorescein succinimidyl ester (CFSE) labeled healthy donor PBMC cells, stimulated with CD3-microbeads at 1.3 beads/cell ratio. After 4-5 days of stimulation, T cell proliferation was determined by flow cytometric analysis of CFSE dilution in corresponding CD4^+^ or CD8^+^ subsets. Treg suppressive function was calculated by the Area Under Curve (AUC) method as described (10). In brief, the rates of division for a range of Treg/responders dilutions were calculated as a % of suppression: [(% of divisions without Treg - % of divisions in current ratio)/% of divisions without Treg]*100. The percentages of suppression for all Treg ratios were evaluated in GraphPad Prism to calculate areas under their suppression curves, using 0 (1/1 ratio), 1 (1/2), 2 (1/4), 3 (1/8) and 4 (1/16) as y-values. Resulted total areas reflect the aggregated suppressive function of Treg over all tested Treg/responders ratios, where higher AUC values indicate stronger suppressive capability of Tregs.

To prepare healthy donor responder cells for suppression assays, one PBMC sample was aliquoted to >200 vials, cryopreserved as described (10), and used as responder cells for each suppression assay to avoid artefacts due to individual variability of human responder cells. Using healthy donor responders in our suppression assays also allowed us to assess patient Treg function independent of possible abnormalities of sarcoidosis T cells, B cells or myeloid cells. Small aliquots of isolated Tregs were cryopreserved to quantitate FOXP3 expression (Treg purity) in isolated cells by flow cytometry. When the number of isolated Tregs allowed, we also performed suppression assays using PBMCs from a second healthy donor, evaluated Treg stability and survival after the suppression assay, tested Treg capacity to suppress cytokine production in healthy donor PBMCs, and evaluated cytokine expression by stable Tregs and by ex-T regulatory cells (ex-Tregs). After suppression assay, dead Tregs were defined as CFSE^-^CD4^+^Ghost^+^FOXP3^+^ and FOXP3^-^ cells; ex-Tregs were defined as CFSE^-^CD4^+^Ghost^-^FOXP3^-^; live stable Tregs were defined as CFSE^-^CD4^+^Ghost^-^FOXP3^+^. Therefore, the stability of Tregs was defined by sustained FOXP3 expression (post-assay) in live cells, while survival was studied by evaluation of (recent) Ghost^+^ dead vs. alive Ghost^-^Tregs.

To evaluate cytokine production, Tregs and PBMCs were mixed 1/1, stimulated overnight with CD3-microbeads, then stimulated with a cocktail of phorbol myristate acetate (3 ng/ml) and ionomycin (1 µM) + Monensin (Biolegend) for 4 hours, and then evaluated by flow cytometry.

### 2.6 Flow cytometry

We randomly selected 3-5 cryopreserved sarcoidosis samples to evaluate different sets of markers in 1-4 flow cytometry panels in one experiment, and for each panel, we also included 1-3 healthy donors PBMC samples. We used additional controls for antibody performance in groups of randomly selected sets of patient samples, with the same healthy donor PBMC aliquots that were used in previous experiments. To control for cryopreservation artefacts, donor PBMC samples were stained for the same set of tested markers before and after cryopreservation. To control for fixation and permeabilization artifacts, expression of tested markers was evaluated in non-fixed cells (without co-staining for FOXP3, CTLA-4, Helios or cytokines) and after fixation. Each cell marker for non-stimulated cells was evaluated using at least 6 different samples from the donor and patient groups. Markers that demonstrated any differences in the initial set of experiments were then evaluated using a larger number of samples.

Healthy donor and patient PBMC samples were evaluated for CD4, CD8, FOXP3, CD45RA, CD45RO, Ki-67, CD39, CTLA-4 and CXCR5 expression. Aliquots of isolated Tregs from healthy donors and sarcoid patients were stained for live/dead fixable reagent, CD4 and FOXP3 expression to control Treg purity after isolation (**Supplementary Figures S1F, S2F**), and for Helios expression.

**Figure 1:**
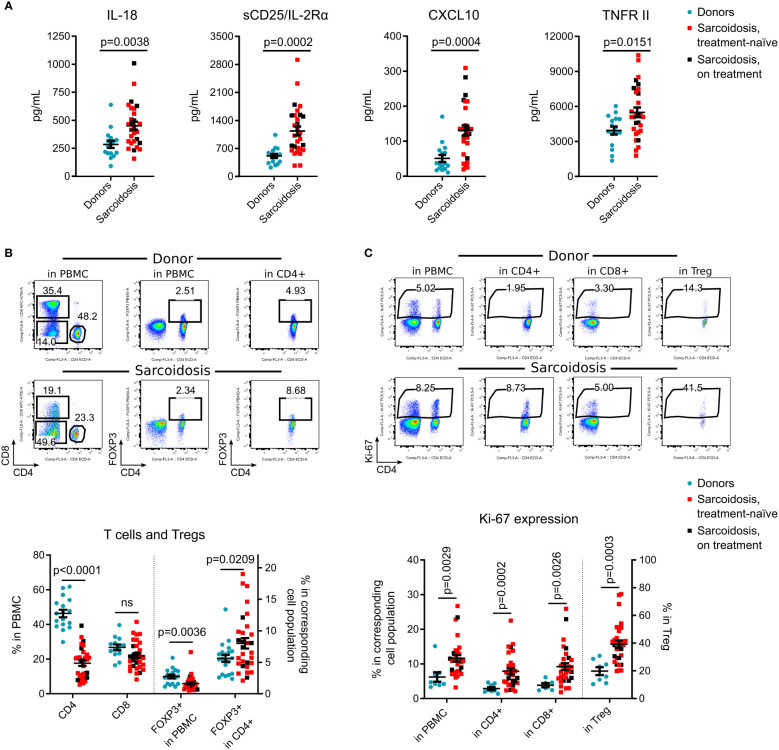
Immune system alterations in sarcoidosis. **(A)**, Plasma levels of IL-18, sCD25/IL-2Rα, CXCL10 and TNFR II were evaluated by Luminex assay in 29 sarcoidosis patients and 16 donors, matched by age, gender, race, and smoking history, **Supplementary Table S2**. Additional Luminex data are presented in **Supplementary Figure S2A**. **(B)**, Percent of CD4^+^ and CD8^+^ T cells, and % of CD4^+^ FOXP3^+^ Tregs within all viable PMBCs and within CD4^+^ subsets were evaluated by flow cytometry. Representative dot plots (top) and corresponding statistics (bottom) are presented. The number of samples evaluated for each group (median, IQR): donors: 17, 14-19.25; sarcoidosis: 30. **(C)**, Ki-67 expression was evaluated in all viable PBMC, in CD4^+^ and CD8^+^ T cells, and in CD4^+^FOXP3^+^ Tregs by flow cytometry. Representative dot plots (top) and corresponding statistics (bottom) are presented. The number of samples: donors: 8, 6.5-8, sarcoidosis: 30. Additional flow cytometry data are shown in **Supplementary Figures S2B-E**. Blue dots represent donors, red squares illustrate data for treatment-naïve sarcoidosis patients, and black squares illustrate sarcoidosis patients receiving treatment. **(A, B)** - unpaired T test; **(C)** - Mann-Whitney test. Graphs show p values for all sarcoidosis samples. Corresponding results for the analysis of treatment-naïve subset versus donors: **(A)** p = 0.006 for IL-18, p = 0.001 for sCD25, p = 0.001 for CXCL10 and p= 0.0299 for TNFR II, unpaired T test. **(B)** p <0.0001 for CD4, p = 0.144 (not significant) for CD8, p = 0.009 for FOXP3^+^ in PBMC and p = 0.033 for FOXP3^+^ in CD4, unpaired T test. **(C)** p = 0.007 for Ki-67^+^ in PBMC, p <0.0001 for Ki-67^+^ in CD4^+^, p = 0.008 for Ki-67^+^ in CD8^+^ and p <0.0001 for Ki-67^+^ in Treg, Mann-Whitney test.

We used a live/dead fixable reagent (**Supplementary Table S3**), then applied Fc blocking reagent (Human TruStain FcX, Biolegend) for 5-10 minutes at room temperature, then stained for surface markers for 40-50 minutes at 4°C in pre-titrated concentrations, then performed Fixation/Permeabilization using Transcription Factor Buffer Set (BD Biosciences) and stained for intranuclear and intracellular antibodies (cytokines, FOXP3, Ki-67, Helios, CTLA-4) for 60 minutes at 4°C. We evaluated cells using CytoFLEX and analyzed data with FlowJo. Compensation was performed using single stains and fluorescent minus one (FMO) controls. We applied gating on cells negative for live/dead fixable reagent (in all experiments) and also gated on CD45^hi+^ population (in some experiments) to exclude dead, apoptotic and non-hematopoietic cells to markedly decrease non-specific signals. Further gating strategies for particular markers and examples of dot plots are shown in **Figures 1B, C**; **Supplementary Figures S1A–G**. Details of flow cytometry antibodies are listed in **Supplementary Table S3**.

### 2.7 Luminex

Cryopreserved plasma samples were evaluated by custom Human Magnetic Luminex Assay (R&D, LXSAHM-14), according to manufacturer instruction. Our custom Human Magnetic Luminex Assay included reagents to evaluate sCD25/IL-2Rα, CXCL10/IP-10, IFNγ, IL-1β, IL-10, IL-12 p70, IL-17A, IL-18, IL-2, IL-6, TNF RI, TNF RII, TNFα and Vitamin D binding peptide. Levels of IFNγ, IL-10 and IL-2 were below detection limits for most plasma samples and were not included in our results.

*Human Treg-Specific Demethylated Region (TSDR) FOXP3 methylation assay* was performed as described (11). In brief, DNA was isolated from Tregs and digested with two restriction enzymes, one methylation-sensitive enzyme and one methylation dependent enzyme. The following day, we amplified four products (no enzymes product served as the “no digestion” control, both enzymes as the “maximal digestion” control, and two products had each enzyme separately) with custom primers for human TSDR FOXP3 (EpiTect II DNA Methylation Enzyme Kit, Qiagen, formerly SABiosciences). Cycle threshold qPCR data in triplicates for 4 DNA products were used to evaluate percent of demethylated and methylated TSDR, according to manufacturer’s instruction.

### 2.8 Statistical analysis

Treg suppressive function was adjusted for FOXP3 purity of isolated Treg (described in (12) and detailed in **Supplementary Methods**). We applied parametric tests for normally distributed data and non-parametric tests otherwise; all tests are indicated in Figure Legends. For regression analysis, we performed univariant analyses of all clinical and immunological variables, and carried forward variables with an alpha <0.15 for differences regarding treatment status at one year follow-up (detailed in **Supplementary Materials**). K-fold cross validation was performed, and data in the main text are reported for the validation set. Data are shown as mean ± SEM. A two-tailed p value of <0.05 was considered statistically significant. We used GraphPad Prism 6.0 and SPSS 17.0. We used the STROBE cohort reporting guidelines (13).

## 3 Results

### 3.1 Clinical features of sarcoidosis

Of the 32 subjects enrolled, two had negative or non-diagnostic biopsies and were excluded from further analysis. The cohort was comprised of 16 males and 14 females, with an average age of 48 ± 1.9 years, and 27% of subjects were Black (**Table 1**). Sixty percent of patients (18/30) never smoked. Obesity was a common co-morbidity (12/30). Otherwise, subjects were largely free from common comorbidities which often contribute to pulmonary symptoms or functional limitations. Most (21/30) had at least one pulmonary symptom, which included, in order of frequency, cough, non-trivial dyspnea, wheezing, chest tightness, and chest pain; no subject reported sputum production or hemoptysis. Eighteen subjects had recently diagnosed disease (disease duration < 1 year) and six had a disease between 1 and 3 years, and for six the duration of disease was > 3 years. Outcome data are missing for three subjects who were lost to clinical follow-up for unknown reasons. At the time of enrollment, most subjects (n = 22) were treatment-naïve, while eight received therapy with systemic corticosteroids, Acthar Gel, or methotrexate (**Table 1**). At follow-up, 59% of treatment-naïve subjects still did not require therapy, while 75% of subjects on therapy at enrollment, had been able to discontinue medication within a year (**Table 1**).

Of those with CT imaging available for review (n=28), nineteen subjects had radiographic evidence of thoracic lymphadenopathy (LAN) (**Table 1**). All but three subjects had pulmonary function testing performed near the time of study entry; of those, one-third had a reduced forced vital capacity (<80% of predicted). In addition to pulmonary involvement, 15 subjects had at least one other documented site of disease, and 13 endorsed systemic symptoms.

### 3.2 Immune system alterations in sarcoidosis

Compared to healthy donors, subjects with sarcoidosis had significantly higher levels of circulating IL-18, CXCL10, sIL-2R/CD25 and TNFR II (**Figure 1A**), but not other tested cytokines (**Supplementary Figure S2A**). Within PBMC, the CD4^+^ subset was considerably decreased in sarcoidosis samples. While the percentage of Tregs, as a fraction of PBMCs, also was significantly reduced, the percentage of Tregs in CD4^+^ cells was increased (**Figure 1B**).

Ki-67, a marker of recent cell division, was upregulated in sarcoidosis PBMCs, in CD4^+^ and CD8^+^ T cells, and most substantially in Tregs (**Figure 1C**), suggesting ongoing clonal activation of T cells with compensatory division of Tregs. In contrast to previously reported data (4, 14, 15), we found that the CD45RO effector/memory subset in sarcoidosis Tregs was decreased, with no differences in expression of CTLA-4 and CD39 (**Supplementary Figures S2B–D**). CXCR5 was upregulated in sarcoidosis Tregs and CD8^+^ cells, but CD4^+^CXCR5^+^ T follicular helper cell numbers were not affected (**Supplementary Figure S2E**).

In a subgroup analysis of treatment-naïve subjects vs. healthy donors, similar results were found for all reported markers (**Figures 1A–C**). Overall, treatment-naïve patients as well as subjects receiving systemic corticosteroids and/or methotrexate, differed from healthy donors with higher levels of IL- 18, sCD25, CXCL10, TNFRII, with lower counts of CD4^+^ T cells but more Tregs within the CD4^+^ subset, and an increased rate of cellular divisions *in vivo*.

### 3.3 Tregs in sarcoidosis

**Figure 2:**
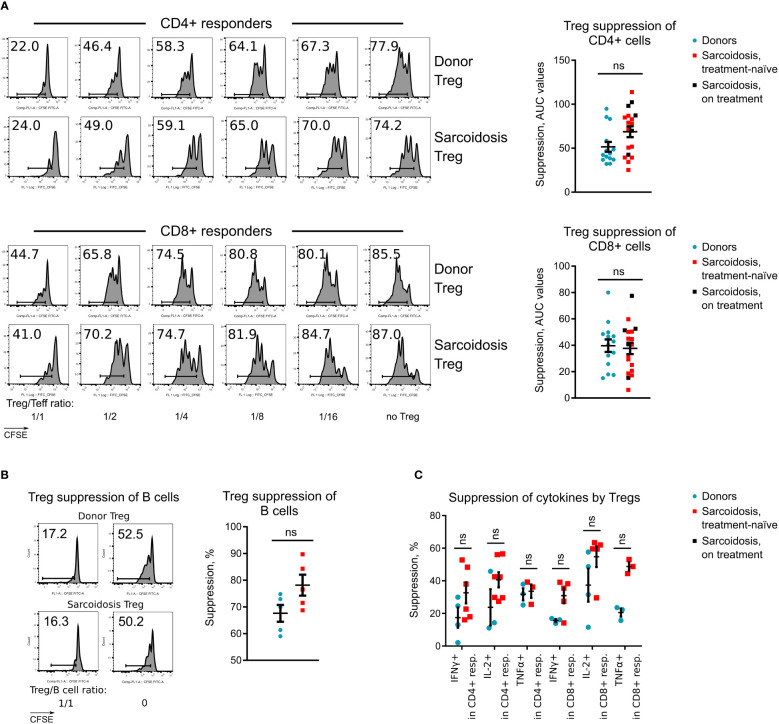
Treg suppressive function in sarcoidosis, **(A)**, Treg suppressive function was evaluated in 14 healthy donors and 20 sarcoidosis patients. Representative CFSE plots for CD4^+^ and CD8^+^ T cell responders (left) and statistics of Treg function adjusted for FOXP3^+^ Treg purity after isolation (right) are shown. Additional data for Treg suppressive function are presented at **Supplementary Figures S2F, G**. **(B)**, Capability of Tregs to suppress CD4^+^ T-helper dependent divisions of healthy donor B cells was evaluated in 5 healthy donors and 5 sarcoidosis patients. CFSE plots for CD19^+^ B cell divisions (left) and statistics (right) of Treg function are shown. **(C)**, Capability of Tregs to suppress cytokine productions by healthy donor CD4^+^ and CD8^+^ T cells, stimulated overnight with CD3-microbeads and stimulated the following day with phorbol myristate acetate + ionomycin is shown. The number of samples evaluated for each group (median, IQR): donors 3, 3-4, sarcoidosis 5, 3-6.25. Blue dots represent donors, red squares illustrate data for treatment-naïve sarcoidosis patients, and black squares illustrate sarcoidosis patients receiving treatment. **(A, B)** - Mann-Whitney test; **(C)** - multiple T-tests with Holm-Sidak correction. ns, non-significant. In A, graphs show p values for all sarcoidosis samples. Corresponding results for the analysis of treatment-naïve subset: p = 0.071 (not significant) for CD4^+^ responders and p = 0.955 (not significant) for CD8^+^ responders, Mann-Whitney test.

To evaluate Treg suppressive function without confounding effects of subject responder and antigen-presenting cells, and to decrease variabilities in results due to differences in FOXP3 purity of isolated Tregs, cryopreserved aliquots of the same healthy donor PBMC sample were used as responders, and suppression assay results were adjusted for FOXP3^+^ Treg purity (**Supplementary Methods**). Sarcoid Tregs suppressed divisions of CD4^+^ and CD8^+^ responders equally well to healthy donor Tregs (**Figure 2A**; **Supplementary Figures S2F, G**). Same data were observed for Tregs isolated from treatment-naïve subjects (**Figure 2A**). We also found no differences in the ability of Tregs to control the expansion of B cells that received stimulatory signals from CD4^+^ helper cells (**Figure 2B**). The ability of sarcoid Tregs to suppress cytokine production was also normal (**Figure 2C**).

**Figure 3:**
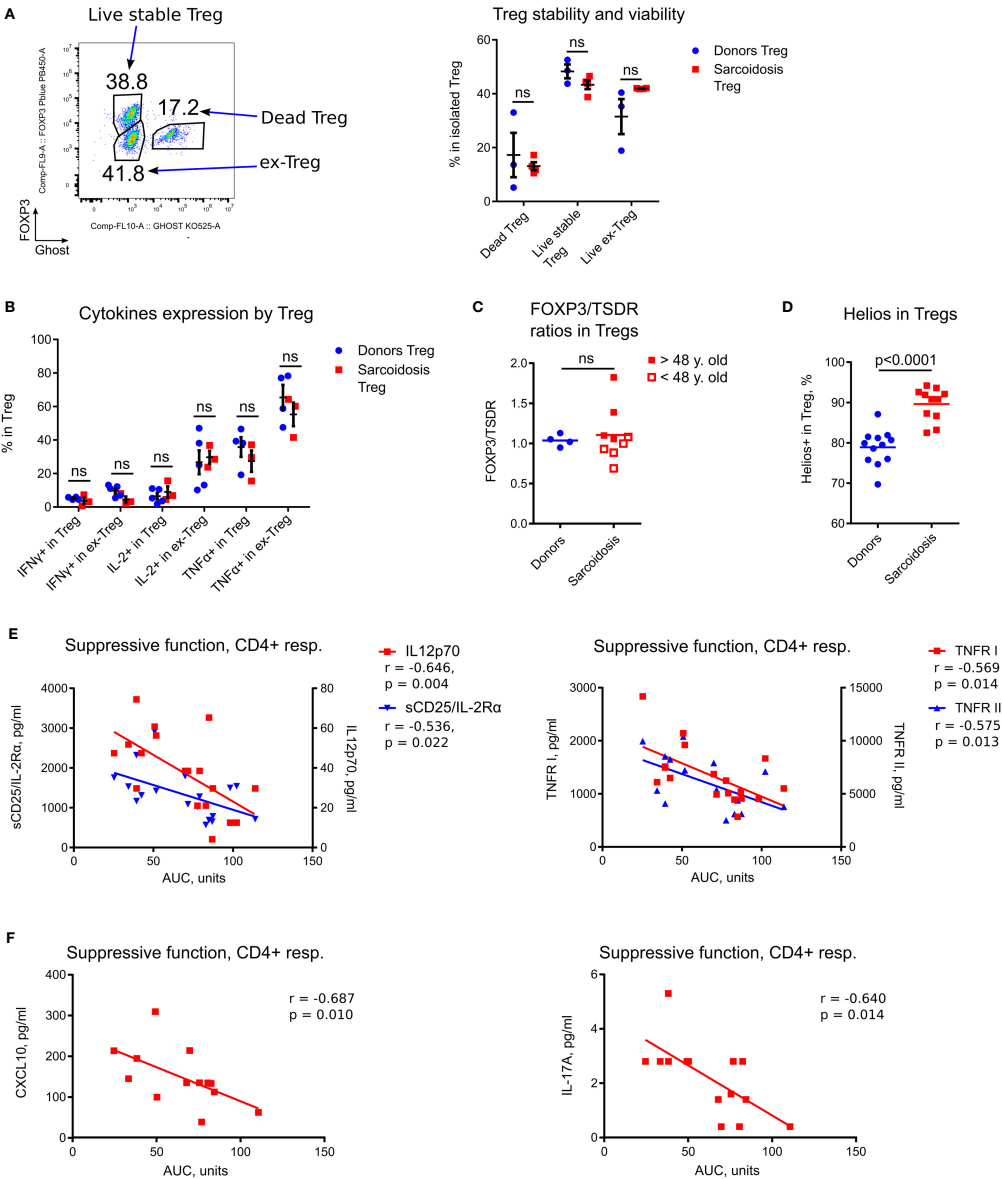
In depth analysis of Tregs and correlations of Treg function with inflammatory markers. **(A)** Evaluation of Treg stability and viability was performed in 3 donor and 4 sarcoidosis samples. Dead Tregs: CFSE^-^CD4^+^Ghost^+^FOXP3^+^ and FOXP3^-^ cells; ex-Tregs: CFSE^-^CD4^+^Ghost^-^FOXP3^-^; live stable Tregs: CFSE^-^CD4^+^Ghost^-^FOXP3^+^. Therefore, the stability of Tregs was defined by sustained FOXP3 expression (post-assay) in live cells, while survival was studied by evaluation of (recent) dead vs. alive Tregs. Representative plot (left) and corresponding statistics (right) are shown. **(B)**, Expression of inflammatory cytokines in Tregs was evaluated in assays as described in **Figure 2C**. CFSE^-^CD4^+^Ghost^-^ viable Tregs were gated according to FOXP3^+^ expression into stable (FOXP3^+^) Treg and ex-Treg (FOXP3^-^). The number of samples evaluated for each group (median, IQR): donors 4.5, 4-5, sarcoidosis 3. **(C)**, Ratios of FOXP3^+^ to TSDR FOXP3 demethylation in isolated Tregs were evaluated in 9 sarcoidosis patients and in 4 healthy donors, matched by gender and age. Additionally, sarcoidosis Treg samples were divided into two groups according to median age (to approximate levels of thymic involution). Samples from older subjects (filled squares) had significantly higher FOXP3/TSDR ratios vs. Treg samples from younger subjects (hollow squares), p = 0.0317. **(D)**, Helios expression in Tregs was evaluated in 12 donors and 11 sarcoidosis samples by flow cytometry. **(E)**, Treg suppressive function in sarcoidosis was inversely correlated with expression of sCD25/IL-2Rα and IL-12p70 (left) and TNFR I and II (right) in plasma, 18 samples evaluated. **(F)**, Treg suppressive function in treatment-naïve patients was inversely correlated with expression of CXCL10 (left) and IL- 17A (right) in plasma, 13 and 14 samples were evaluated, correspondingly. **(A, B)** - multiple T-tests with Holm-Sidak correction; **(C, D)** - Mann-Whitney test; **(E, F)** - Spearman’s correlation. ns, non-significant.

As sarcoid Tregs have been reported to be less viable and prone to apoptosis (14), we studied Treg survival and stability, and found them to be unimpaired (**Figure 3A**). We then evaluated the production of inflammatory cytokines by stable Tregs (CD4^+^FOXP3^+^) and by ex-Treg (CD4^+^FOXP3^-^ cells that initially were FOXP3^+^ after isolation). Stable sarcoid Tregs produced trivial amounts of IFNγ and IL- 2, as expected, while ex-Tregs started to produce IL-2, albeit at the same levels as donor ex-Tregs. All types of Tregs produced comparable amount of TNFα (**Figure 3B**).

We used a TSDR assay in combination with an assessment of FOXP3 protein expression, as previously described (16), to evaluate two subpopulations of Tregs: thymic derived Tregs (tTreg) and adaptive or induced Tregs (iTreg). The ratios of iTregs to tTregs were similar for sarcoidosis and donor samples (**Figure 3C**). Similar to our previous findings (11), we observed an age-related increase in the fraction of iTreg attributable to thymic involution (**Figure 3C**). Notably, sarcoid Tregs demonstrated upregulated Helios expression by flow cytometry (**Figure 3D**) which corresponded to upregulated Ki- 67^+^, further confirming the role of Helios as a marker for T cell activation and division, as we have previously reported (17), and arguing against Helios as a marker of tTregs.

### 3.4 Inflammatory cytokines and Ki-67 expression as potential markers of Treg function *in vivo*

To evaluate if Treg function *in vitro* reflects inflammatory abnormalities or immune features *in vivo*, we compared sarcoid Treg function and Treg numbers with levels of inflammatory markers and Ki-67 expression. We found that Treg suppressive function for CD4^+^ responder cells inversely correlated with levels of the inflammatory markers sCD25, IL-12p70, TNFR I, TNFR II (**Figure 3E**), and with Ki-67^+^ expression (**Table 2**; **Supplementary Figure S3A**), suggesting that the suppressive capabilities of sarcoid Tregs measured *in vitro* reflect *in vivo* capacities. We observed similar though not significant trends for correlations between Treg suppressive function *in vitro* and KI-67^+^ expression in CD8^+^ T cells and other PBMCs (**Supplementary Figures S3A, B**). Similar findings were observed in the subgroup of treatment-naïve subjects, but additionally, their Treg suppressive function for CD4^+^ responders negatively correlated with plasma levels of CXCL10 and IL-17A (**Figure 3F**).

**Table 2:** Correlation matrix of inflammatory markers in plasma, Treg suppressive function, number of Tregs within the CD4^+^ subset, and Ki-67^+^ expression in the sarcoidosis cohort.

Plasma and cellular markers		Suppr function, CD4+ resp.	IL18 in plasma	IL1β in plasma	TNFα in plasma	IL-6 in plasma	CXCL 10 in plasma	IL-17a in plasma	sCD25/IL2Ra in plasma	IL12 p70 in plasma	TNFR II in plasma	TNFR I in plasma	FOXP3+ Treg in CD4+	Ki67+ in PBMC	Ki67+ in CD4+	Ki67+ in CD8+	Ki67+ in CD4-CD8- non T cells
IL18 in plasma	r	.194	1.000	.231	.395*	-.072	.465*	-.158	.502**	-.126	.604**	.378*	.522**	.430*	.249	.362	.322
	Sig.	.456	.	.237	.038	.717	.013	.421	.006	.522	.001	.048	.004	.022	.201	.059	.095
	N	17	28	28	28	28	28	28	28	28	28	28	28	28	28	28	28
IL1β in plasma	r	-.440	.231	1.000	.559**	.529**	.084	.483**	.204	.698**	.368*	.242	.161	.370*	.562**	.293	.343
	Sig.	.068	.237	.	.002	.003	.672	.008	.289	.000	.049	.207	.403	.048	.002	.123	.068
	N	18	28	29	28	29	28	29	29	29	29	29	29	29	29	29	29
TNFα in plasma	r	-.316	.395*	.559**	1.000	.486**	.319	.283	.662**	.526**	.594**	.482**	.249	.415*	.514**	.356	.373
	Sig.	.217	.038	.002	.	.009	.098	.145	.000	.004	.001	.009	.202	.028	.005	.063	.050
	N	17	28	28	28	28	28	28	28	28	28	28	28	28	28	28	28
IL-6 in plasma	r	-.395	-.072	.529**	.486**	1.000	.103	.420*	.315	.470*	.181	.388*	.131	.365	.379*	.412*	.348
	Sig.	.105	.717	.003	.009	.	.603	.023	.096	.010	.347	.038	.497	.052	.043	.026	.064
	N	18	28	29	28	29	28	29	29	29	29	29	29	29	29	29	29
CXCL 10 in plasma	r	-.292	.465*	.084	.319	.103	1.000	-.211	.679**	-.144	.456*	.461*	.288	.443*	.217	.358	.333
	Sig.	.256	.013	.672	.098	.603	.	.282	.000	.465	.015	.014	.137	.018	.268	.061	.084
	N	17	28	28	28	28	28	28	28	28	28	28	28	28	28	28	28
IL-17a in plasma	r	-.273	-.158	.483**	.283	.420*	-.211	1.000	.125	.517**	.012	.006	.121	.240	.521**	.175	.168
	Sig.	.274	.421	.008	.145	.023	.282	.	.518	.004	.952	.977	.531	.210	.004	.365	.383
	N	18	28	29	28	29	28	29	29	29	29	29	29	29	29	29	29
sCD25/IL2Ra in plasma	r	-.536*	.502**	.204	.662**	.315	.679**	.125	1.000	.227	.781**	.741**	.552**	.599**	.429*	.535**	.551**
	Sig.	.022	.006	.289	.000	.096	.000	.518	.	.236	.000	.000	.002	.001	.020	.003	.002
	N	18	28	29	28	29	28	29	29	29	29	29	29	29	29	29	29
IL12 p70 in plasma	r	-.646**	-.126	.698**	.526**	.470*	-.144	.517**	.227	1.000	.306	.112	.317	.234	.645**	.256	.265
	Sig.	.004	.522	.000	.004	.010	.465	.004	.236	.	.106	.562	.094	.221	.000	.180	.166
	N	18	28	29	28	29	28	29	29	29	29	29	29	29	29	29	29
TNFR II in plasma	r	-.575*	.604**	.368*	.594**	.181	.456*	.012	.781**	.306	1.000	.785**	.437*	.523**	.388*	.373*	.581**
	Sig.	.013	.001	.049	.001	.347	.015	.952	.000	.106	.	.000	.018	.004	.038	.046	.001
	N	18	28	29	28	29	28	29	29	29	29	29	29	29	29	29	29
TNFR I in plasma	r	-.569*	.378*	.242	.482**	.388*	.461*	.006	.741**	.112	.785**	1.000	.200	.535**	.217	.300	.607**
	Sig.	.014	.048	.207	.009	.038	.014	.977	.000	.562	.000	.	.298	.003	.259	.114	.000
	N	18	28	29	28	29	28	29	29	29	29	29	29	29	29	29	29
FOXP3+ Treg in CD4+	r	-.395	.522**	.161	.249	.131	.288	.121	.552**	.317	.437*	.200	1.000	.485**	.526**	.795**	.397*
	Sig.	.104	.004	.403	.202	.497	.137	.531	.002	.094	.018	.298	.	.007	.003	.000	.030
	N	18	28	29	28	29	28	29	29	29	29	29	30	30	30	30	30
Ki67+ in PBMC	r	-.431	.430*	.370*	.415*	.365	.443*	.240	.599**	.234	.523**	.535**	.485**	1.000	.639**	.645**	.907**
	Sig.	.074	.022	.048	.028	.052	.018	.210	.001	.221	.004	.003	.007	.	.000	.000	.000
	N	18	28	29	28	29	28	29	29	29	29	29	30	30	30	30	30
Ki67+ in CD4+	r	-.641**	.249	.562**	.514**	.379*	.217	.521**	.429*	.645**	.388*	.217	.526**	.639**	1.000	.494**	.441*
	Sig.	.004	.201	.002	.005	.043	.268	.004	.020	.000	.038	.259	.003	.000	.	.006	.015
	N	18	28	29	28	29	28	29	29	29	29	29	30	30	30	30	30
Ki67+ in CD8+	r	-.358	.362	.293	.356	.412*	.358	.175	.535**	.256	.373*	.300	.795**	.645**	.494**	1.000	.583**
	Sig.	.145	.059	.123	.063	.026	.061	.365	.003	.180	.046	.114	.000	.000	.006	.	.001
	N	18	28	29	28	29	28	29	29	29	29	29	30	30	30	30	30
Ki67+ in CD4-CD8- non T cells	r	-.455	.322	.343	.373	.348	.333	.168	.551**	.265	.581**	.607**	.397*	.907**	.441*	.583**	1.000
	Sig.	.058	.095	.068	.050	.064	.084	.383	.002	.166	.001	.000	.030	.000	.015	.001	.
	N	18	28	29	28	29	28	29	29	29	29	29	30	30	30	30	30

Positive significant correlations (Spearman) highlighted at yellow; negative significant correlations highlighted at blue, where significance is adjusted by the Benjamini–Hochberg multiple comparison procedure (18) at p<0.0204, 2-tailed. In treatment-naïve patients, 76% of correlations are the same, with the remaining correlations having the same trends but without significance.

* not adjusted p value is less than 0.05; ** not adjusted p value is less than 0.01.

Most of the tested inflammatory markers in sarcoidosis samples significantly correlated with each other, and also with Ki- 67 expression among different PBMC subsets (**Table 2**). This suggests that insufficient Treg control *in vivo* led to activation of immune cells (i.e. producing more cytokines) along with their increased rate of divisions, reflected by higher Ki-67^+^ expression. Notably, the inflammatory markers in the plasma of healthy donors were much less correlated (**Supplementary Table 4**).

Treg numbers positively correlated with IL-18, sCD25, with TNFR II in plasma and with Ki-67^+^ in all PBMC subsets (**Table 2**), suggesting that increased number of Tregs in sarcoidosis patients may reflect a compensatory effort of the immune system to control ongoing inflammation. Taken together, our findings suggest that Ki-67^+^ in PBMCs and in T cells, and the inflammatory markers IL-12p70, TNFR I, TNFR II, and sCD25 may serve as indirect measures of Treg suppressive activity *in vivo*. We have recently reported similar inverse correlation between Ki-67 expression and Treg suppressive function in lung transplant recipients (12).

### 3.5 Association of immune markers with clinical features

**Figure 4:**
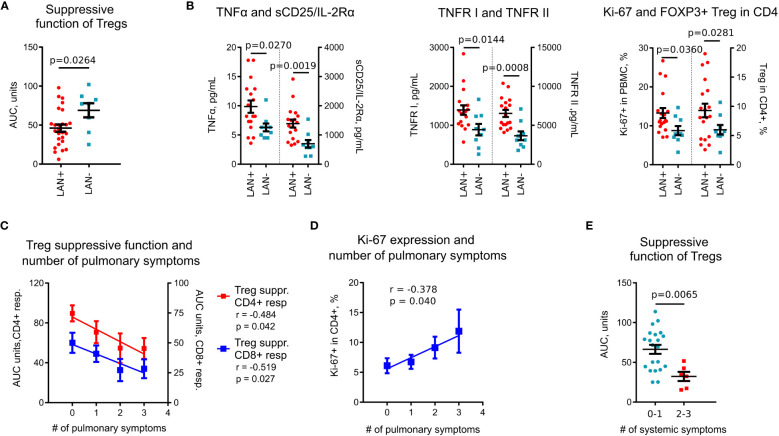
Association of immune markers with clinical features and Tregs in sarcoidosis. **(A)**, Treg suppressive function was lower in patients with thoracic lymphadenopathy (LAN+) compared to patients without thoracic lymphadenopathy (LAN-). Data for CD4^+^ and CD8^+^ T cell responders in Treg suppression assays are combined. **(B)**, Plasma levels of TNFα, sCD25/IL-2Rα, and TNFR I and II, evaluated by Luminex assay, were higher in LAN+ (n =18) compared to LAN- (n = 9) patients. Expression of Ki-67 in PBMCs and Treg numbers in CD4^+^ T cells, evaluated by flow cytometry, were compared between LAN+ (n = 19) and LAN- (n = 9) patients. **(C)**, Treg suppressive function in sarcoidosis patients was inversely correlated with the number of pulmonary symptoms, n = 18. **(D)**, Ki-67 expression was positively correlated with the number of pulmonary symptoms, n = 30. **(E)**, Treg suppressive function was decreased in patients with more than one systemic symptom (n = 6) compared to patients with 0-1 systemic symptoms (n = 21). Data for CD4^+^ and CD8^+^ responders in Treg suppression assays are combined. **(A)** - unpaired T test; **(B)** (TNFα, sCD25, TNFR I and II, Ki-67 in PBMC) - Mann-Whitney test; **(B)** (Tregs in CD4^+^ cells) - unpaired T test with Welch’s correction; **(C, D)** - Spearman’s correlation; **(E)** – Mann-Whitney test.

With the hypothesis that sub-phenotypes of pulmonary sarcoidosis have their basis in distinct immunologic features, we compared immunological variables against clinical data. Among imaging features, Treg suppressive function was reduced in subjects with thoracic lymphadenopathy (LAN) (**Figure 4A**). Subjects with LAN also had significantly increased levels of plasma TNFα, sCD25, TNFR I and II, had more Tregs, and upregulated Ki-67 expression (**Figure 4B**). In a subgroup analysis of treatment-naïve subjects, these findings were similar, with the additional finding that those with LAN, had increased levels of CXCL10 compared to subset without LAN (145.8 pg/ml vs. 79.6 pg/ml, p = 0.024, Mann Whitney test).

Symptoms also were associated with immunologic markers indicating poorly controlled systemic inflammatory activity. Treg suppressive function had a strong negative correlation (**Figure 4C**), while Ki-67 positively correlated (**Figure 4D**) with the number of pulmonary symptoms. Treg function was also reduced in subjects with a high burden of systemic symptoms (**Figure 4E**).

Therefore, we have identified a set of immunological parameters including Treg suppressive function, TNFα, TNFR I and II, sCD25 and Ki-67^+^ that are associated with clinical features in sarcoidosis. Notably, a set of these inflammatory markers was also increased in sarcoidosis subjects in comparison with healthy donors, and most of them were negatively correlated with Treg suppressive function.

### 3.6 Association of immune markers with outcomes of sarcoidosis

**Figure 5:**
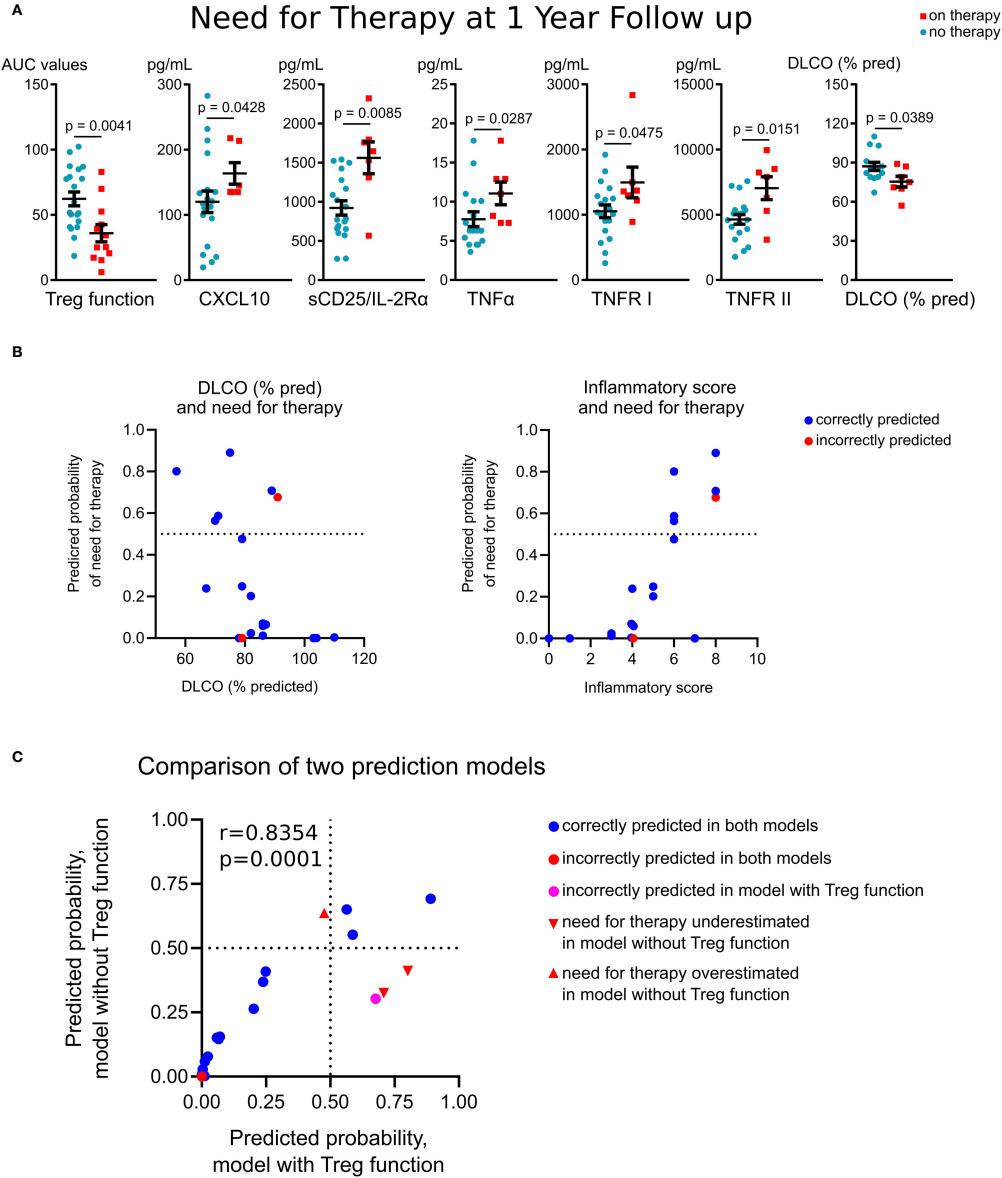
Association of Treg function and immune markers with outcomes in sarcoidosis. **(A)**, Treg suppressive function, evaluated at study entry, was significantly reduced in subjects who required treatment at one year follow-up (n = 6) compared to patients who did not require treatment (n = 10). Plasma levels of CXCL10 (n = 6 on therapy, n = 19 no therapy required) and sCD25/IL-2Rα, TNFR I and II (n = 7 on therapy, n = 19 no therapy required) were compared. DLCO at enrolment also was associated with treatment need at follow-up (n = 7 on therapy, n = 14 no therapy required). **(B)**, Six probit regression models with k-Fold cross validation were run using need for therapy as the dependent variable and the inflammatory score (derived from Treg function, sCD25, TNFR I and II, TNFα, CXCL10 and Ki-67^+^ in PBMC) and DLCO (% pred) as predictors. The fitted regression equation was: probit (P_on therapy)_ = 7.17*inflammatory score – 9.76*DLCO%. Both coefficients were significant at p = 0.007 and p = 0.002 levels, respectively. The overall regression was statistically significant (likelihood ratio Chi-Square χ^2^(2) = 10.96, p = 0.004). Correctly classified (90%, blue dots) and misclassified (red dots) cases are shown with predictive probability of need for therapy for validation set of patients as a function of DLCO, % pred (left) and the inflammatory score (right). Further details are provided in **Supplementary Table S5**. **(C)**, Comparison of probit models with and without Treg function as a part of inflammatory scores are detailed in **Supplementary Table S6**. **(A)** (except DLCO) – Mann-Whitney test; **(A)** (DLCO) – unpaired T test, **(C)** – Pearson’s correlation.

To evaluate if immunologic measures at study entry were associated with long-term outcomes, we compared variables with need for treatment at one year follow-up. We defined disease status by treatment need rather than other objective and subjective measures, as treatment can stabilize pulmonary function, making a decline in pulmonary function tests values an insensitive marker of continued disease activity, and pulmonary symptoms poorly differentiate between inflammatory and fibrotic phases of disease. Baseline Treg suppressive function was significantly reduced in subjects who required treatment at one year follow-up (**Figure 5A**). These subjects also had higher baseline plasma levels of CXCL10, sCD25, TNFR I and II and significantly lower baseline values of the diffusion capacity of the lungs for carbon monoxide (DLCO, % predicted) (**Figure 5A**). In a subgroup analysis of treatment-naïve subjects, these findings were similar, with those needing therapy at one year follow-up, demonstrating reduced Treg function and higher levels of CXCL10, sCD25 and TNFR II at baseline, with the additional finding of enhanced division rate of PBMCs at baseline (14.2% vs. 9.2% were Ki-67^+^, p=0.027).

A regression analysis with K-fold cross-validation was performed to ascertain the effects of immunological markers and clinical differences at study entry on the likelihood that subjects required therapy at one year follow-up. The combination of two and more variables caused collinearity due to strong correlations of inflammatory markers with each other and with Treg suppressive function (**Table 2**). To overcome this issue, we combined Treg function (for CD4^+^ and CD8^+^ responders separately), and levels of sCD25, TNFR I and II, TNFα, CXCL10 and Ki-67^+^in PBMC cells into a single score variable. Thus, each inflammatory variable was scored 1 if increased compared to the mean value of healthy donors, or 0 otherwise. For Treg function, cases with impaired suppression were scored as 1, or 0 otherwise. The resulting “summary inflammatory score”, ranging 0 to 8, was used in the regression models. Six probit regression models with k-Fold cross validation were run (size of training sets were 16, 17, 15, 18, 17 and 16 observations, correspondingly). The resulted probit model (**Figure 5B**; **Supplementary Table S5**) showed an increased probability of need for therapy in patients with a lower DLCO and higher inflammatory scores at baseline, and correctly classified 90% (18/20) of cases in the validation set (**Figure 5B**). Of the two misclassified patients, one had increased expression of all inflammatory markers, high Ki-67 expression and impaired Treg function, and therefore had a high 67.6% prediction for need for sarcoid therapy, but this patient was on immunosuppression for autoimmune disease (#10, **Table 1**). For treatment-naïve patients with less than 3 years of disease duration, the model correctly predicted need for therapy in 85.7% cases (**Supplementary Table S5**, the same two subjects were misclassified).

As evaluation of Treg function may be challenging for clinical laboratories, we developed an alternative model to indirectly assess Treg functionality. The best performance was found for the same combination of predictive variables: DLCO (% pred.) and inflammatory scores, but without inclusion of Treg function in the latter (these inflammatory scores ranged 0-6, **Supplementary Table S6**). Both this and Treg-inclusive models correlated with each other, but the absence of Treg function led to the misclassification of four patients, and the overall correct prediction accuracy in the validation set fell to 80% (**Figure 5C**). The most notable effect was a decrease in the predicted accuracy for patients on therapy at one year follow-up: only 50% of these patients were classified correctly (**Supplementary Table S6**). Of the misclassified subjects, two cases incorrectly predicted to not require treatment had impaired Treg function (#11 and #15, **Table 1**; **Figure 5C**), and one case incorrectly predicted as “high risk” had well preserved Treg function (#6, **Table 1**; **Figure 5C**). These data illustrate that Treg function, when assessed directly, is important predictor of disease prognosis and contributes meaningfully to prognostic models. Therefore, according to our data, the model without Treg function cannot be applicable to treatment prediction in clinical practice.

In summary, reduced Treg function, higher levels of plasma CXCR10, sCD25, TNFR I and II, higher Ki-67^+^ levels, and lower DLCO at baseline were associated with worse prognosis in our cohort.

## 4 Discussion

The goal of our study was to perform an in-depth analysis of Tregs in sarcoidosis patients in relation to immune markers and to assess the association of immunologic parameters with disease prognosis. A limited number of studies have previously explored the role of Tregs in sarcoidosis (2–6), with conflicting results of Treg function (2, 3, 5, 6). Conflicting results may be related to the use of autologous responder cells, which are prone to effects of patient T cell abnormalities, the use of a thymidine incorporation assay which cannot distinguish between direct induction of cell death and reduction of proliferation (10), and inconsistently reported or evaluated FOXP3^+^ purity of isolated Tregs. We adjusted Treg suppressive function for FOXP3^+^ Treg purity and used highly controlled experimental conditions with responder cells from the same donor for our analyses. With this approach, we found no impairments in Treg suppressive capabilities *in vitro* to control division and cytokine production by T cells and B cells and no alterations in FOXP3 stability or Treg viability in comparison with healthy donors. In addition, we are the first to evaluate iTreg/tTreg ratios in sarcoidosis Tregs and found no abnormalities, suggesting that the balance of Treg conversion and thymic production of Tregs is not impaired.

Similar to other reports (19–27), we found increased levels of inflammatory cytokines in sarcoidosis. Our findings that a variety of these markers are negatively correlated with Treg function are novel. High Ki-67^+^ expression along with increased levels of inflammatory markers, suggest that sarcoidosis Tregs may be unable to sufficiently control immune activation *in vivo*, despite normal Treg function *in vitro*. Such discrepancies have been previously reported for murine Tregs (28). Another explanation which was not addressed in the current study is the resistance of sarcoid T cells, possibly along with monocytes or other immune cells, to Treg suppression, i.e. the situation where Tregs have no apparent abnormalities, but resulting Treg control is still not efficient.

In our cohort, Treg suppressive function was decreased while inflammatory markers were upregulated in the subgroup of subjects with LAN. These finding are in contrast to the more favorable prognosis for patients without LAN observed by Scadding decades ago, albeit with a different imaging modality between that study and ours (29). However, a recent study on non-sarcoidosis interstitial lung disease found, similar to our results, that LAN was associated with worse clinical measures and outcomes (30).

Prior studies have found that circulating levels of sCD25 and TNF RI and II correlated with disease activity in sarcoidosis (19, 25–27, 31–33), and that Ki-67 expression was increased in sarcoid granulomas (34, 35). To our knowledge we are the first to demonstrate that Ki-67 is altered in the blood in sarcoidosis, with higher expression associated with impaired Treg function and increased levels of inflammatory markers. Together, our findings suggest that a combination of markers including Ki-67 expression may be used for assessing disease activity in sarcoidosis.

There are several important limitations of this study. Our outcome assessment was limited to pulmonary status. To investigate the possible role of Treg function in pulmonary sarcoidosis, our cohort was enriched for patients with findings suggestive of active disease, including pulmonary symptoms attributable to sarcoidosis with nodular features on chest imaging resembling inflammatory changes. However, a gold standard biomarker for sarcoidosis activity is lacking, and some in our cohort may have been in a quiescent phase of disease. Dyspnea was assessed in a binary fashion (present or not). Use of a validated symptom scale could allow for a more granular assessment of dyspnea burden. Another limitation is the relatively small size of our cohort and the single center design which may restrict the generalization of our observations. We utilized two different cohorts of healthy donors for PBMCs and for the plasma to ensure matching plasma samples by age, gender, race and smoking history, which may affect reproducibility of healthy donor’s data. Finally, in the current study we evaluated blood Tregs and levels of circulating cytokines, but not bronchoalveolar lavage cells or immune markers. It still remains to be seen to what extent local features are reflected in peripheral blood.

In conclusion, we have identified a set of inflammatory markers (sCD25, TNFR I and II) and Ki- 67 expression in sarcoidosis which may reflect suppressive capability of FOXP3^+^ Tregs *in vivo*, and therefore serve as indirect measures of Treg suppressive function. In addition, these markers, along with CXCL10 and TNFα, may be useful to identify patients who will require treatment at one year follow-up. In our cohort, direct and indirect measures of impaired Treg function were associated with worse clinical measures and outcomes, suggesting that Treg function contributes to the pathogenesis in sarcoidosis. Further work is needed to determine if manipulation of Treg function could be therapeutically employed to improve clinical outcomes.

## Data availability statement

The raw data supporting the conclusions of this article will be made available by the authors, without undue reservation.

## Ethics statement

The studies involving humans were approved by Institutional Review Board, Upenn #819345. The studies were conducted in accordance with the local legislation and institutional requirements. The participants provided their written informed consent to participate in this study.

## Author contributions

KP: Conceptualization, Data curation, Funding acquisition, Investigation, Resources, Validation, Writing – original draft, Writing – review & editing. WM: Investigation, Validation, Writing – review & editing. WH: Conceptualization, Funding acquisition, Resources, Writing – review & editing. TA: Conceptualization, Data curation, Formal analysis, Investigation, Methodology, Validation, Visualization, Writing – original draft, Writing – review & editing.

## References

[1] Kaiser Y Eklund A Grunewald J. Moving target: shifting the focus to pulmonary sarcoidosis as an autoimmune spectrum disorder. Eur Respir J (2019) 54(1):1802153. doi: 10.1183/13993003.021532018 31000677

[2] Oswald-Richter KA Richmond BW Braun NA Isom J Abraham S Taylor TR. Reversal of global CD4+ subset dysfunction is associated with spontaneous clinical resolution of pulmonary sarcoidosis. J Immunol (2013) 190(11):5446–53. doi: 10.4049/jimmunol.1202891 PMC3660530 23630356

[3] Broos CE van Nimwegen M In 't Veen JC Hoogsteden HC Hendriks RW van den Blink B. Decreased cytotoxic T-lymphocyte antigen 4 expression on regulatory T cells and th17 cells in sarcoidosis: double trouble? Am J Respir Crit Care Med (2015) 192(6):763–5. doi: 10.1164/rccm.201503-0635LE 26371815

[4] Taflin C Miyara M Nochy D Valeyre D Naccache JM Altare F. FoxP3+ regulatory T cells suppress early stages of granuloma formation but have little impact on sarcoidosis lesions. Am J Pathol (2009) 174(2):497–508. doi: 10.2353/ajpath.2009.080580 19147826 PMC2630558

[5] Rappl G Pabst S Riemann D Schmidt A Wickenhauser C Schutte W. Regulatory T cells with reduced repressor capacities are extensively amplified in pulmonary sarcoid lesions and sustain granuloma formation. Clin Immunol (2011) 140(1):71–83. doi: 10.1016/j.clim.2011.03.015 21482483

[6] Miyara M Amoura Z Parizot C Badoual C Dorgham K Trad S. The immune paradox of sarcoidosis and regulatory T cells. J Exp Med (2006) 203(2):359–70. doi: 10.1084/jem.20050648 PMC2118208 16432251

[7] Harris PA Taylor R Thielke R Payne J Gonzalez N Conde JG. Research electronic data capture (REDCap)–a metadata-driven methodology and workflow process for providing translational research informatics support. J BioMed Inform (2009) 42(2):377–81. doi: 10.1016/j.jbi.2008.08.010 PMC2700030 18929686

[8] Miller MR. General considerations for lung function testing. Eur Respir J (2005) 26(1):153–61. doi: 10.1183/09031936.05.00034505 15994402

[9] Judson MA Preston S Hu K Zhang R Jou S Modi A. Quantifying the relationship between symptoms at presentation and the prognosis of sarcoidosis. Respir Med (2019) 152:14–9. doi: 10.1016/j.rmed.2019.03.012 31128604

[10] Akimova T Levine MH Beier UH Hancock WW. Standardization, evaluation, and area-under-curve analysis of human and murine treg suppressive function. Methods Mol Biol (2016) 1371:43–78. doi: 10.1007/978-1-4939-3139-2_4 26530794 PMC5554116

[11] Akimova T Zhang T Negorev D Singhal S Stadanlick J Rao A. Human lung tumor FOXP3+ Tregs upregulate four "Treg-locking" transcription factors. JCI Insight (2017) 2(16):e94075. doi: 10.1172/jci.insight.94075 28814673 PMC5621877

[12] Akimova T Zhang T Christensen LM Wang Z Han R Negorev D. Obesity-related IL-18 impairs treg function and promotes lung ischemia-reperfusion injury. Am J Respir Crit Care Med (2021) 204(9):1060–74. doi: 10.1164/rccm.202012-4306OC PMC8663013 34346860

[13] von Elm E Altman DG Egger M Pocock SJ Gotzsche PC Vandenbroucke JP. The Strengthening the Reporting of Observational Studies in Epidemiology (STROBE) statement: guidelines for reporting observational studies. J Clin Epidemiol (2008) 61(4):344–9. doi: 10.1016/j.jclinepi.2007.11.008 18313558

[14] Broos CE Van Nimwegen M Kleinjan A Ten Berge B Muskens F In ‘T Veen JCCM. Impaired survival of regulatory T cells in pulmonary sarcoidosis. Respir Res (2015) 16(1):108. doi: 10.1186/s12931-015-0265-8 26376720 PMC4574219

[15] Kudryavtsev IV Lazareva NM Baranova OP Golovkin AS Isakov DV Serebriakova MK. CD39+ regulatory T cells in pulmonary sarcoidosis and Lofgren's syndrome. Med Immunol (Russia) (2019) 21:467–78. doi: 10.15789/1563-0625-2019-3-467-478

[16] Akimova T Kamath BM Goebel JW Meyers KE Rand EB Hawkins A. Differing effects of rapamycin or calcineurin inhibitor on T-regulatory cells in pediatric liver and kidney transplant recipients. Am J Transplant (2012) 12(12):3449–61. doi: 10.1111/j.1600-6143.2012.04269.x PMC3513508 22994804

[17] Akimova T Beier UH Wang L Levine MH Hancock WW. Helios expression is a marker of T cell activation and proliferation. PLoS One (2011) 6(8):e24226. doi: 10.1371/journal.pone.0024226 21918685 PMC3168881

[18] Lewis C. Multiple Comparisons. In: Peterson P Baker E McGaw B, editors. International Encyclopedia of Education, 3rd ed. Oxford, England: Elsevier (2010). p. 312–8.

[19] Thi Hong Nguyen C Kambe N Kishimoto I Ueda-Hayakawa I Okamoto H. Serum soluble interleukin-2 receptor level is more sensitive than angiotensin-converting enzyme or lysozyme for diagnosis of sarcoidosis and may be a marker of multiple organ involvement. J Dermatol (2017) 44(7):789–97. doi: 10.1111/1346-8138.13792 28295528

[20] Eurelings LEM Miedema JR Dalm V van Daele PLA van Hagen PM van Laar JAM. Sensitivity and specificity of serum soluble interleukin-2 receptor for diagnosing sarcoidosis in a population of patients suspected of sarcoidosis. PLoS One (2019) 14(10):e0223897. doi: 10.1371/journal.pone.0223897 31622413 PMC6797090

[21] Su R Nguyen ML Agarwal MR Kirby C Nguyen CP Ramstein J. Interferon-inducible chemokines reflect severity and progression in sarcoidosis. Respir Res (2013) 14:121. doi: 10.1186/1465-9921-14-121 24199653 PMC4176097

[22] Arger NK Ho ME Allen IE Benn BS Woodruff PG Koth LL. CXCL9 and CXCL10 are differentially associated with systemic organ involvement and pulmonary disease severity in sarcoidosis. Respir Med (2020) 161:105822. doi: 10.1016/j.rmed.2019.105822 31783271 PMC7028429

[23] Shigehara K Shijubo N Ohmichi M Yamada G Takahashi R Okamura H. Increased levels of interleukin-18 in patients with pulmonary sarcoidosis. Am J Respir Crit Care Med (2000) 162(5):1979–82. doi: 10.1164/ajrccm.162.5.9911113 11069843

[24] Kieszko R Krawczyk P Jankowska O Chocholska S Krol A Milanowski J. The clinical significance of interleukin 18 assessment in sarcoidosis patients. Respir Med (2007) 101(4):722–8. doi: 10.1016/j.rmed.2006.08.019 17015003

[25] Ziegenhagen MW Fitschen J Martinet N Schlaak M Muller-Quernheim J. Serum level of soluble tumour necrosis factor receptor II (75 kDa) indicates inflammatory activity of sarcoidosis. J Intern Med (2000) 248(1):33–41. doi: 10.1046/j.1365-2796.2000.00685.x 10947879

[26] Kieszko R Krawczyk P Chocholska S Bojarska-Junak A Jankowska O Krol A. Tumor necrosis factor receptors (TNFRs) on T lymphocytes and soluble TNFRs in different clinical courses of sarcoidosis. Respir Med (2007) 101(3):645–54. doi: 10.1016/j.rmed.2006.06.004 16889950

[27] Nakayama T Hashimoto S Amemiya E Horie T. Elevation of plasma-soluble tumour necrosis factor receptors (TNF-R) in sarcoidosis. Clin Exp Immunol (1996) 104(2):318–24. doi: 10.1046/j.1365-2249.1996.13702.x PMC2200429 8625527

[28] Shevach EM. Foxp3(+) T regulatory cells: still many unanswered questions-A perspective after 20 years of study. Front Immunol (2018) 9:1048. doi: 10.3389/fimmu.2018.01048 29868011 PMC5962663

[29] Scadding JG. Prognosis of intrathoracic sarcoidosis in England. A review of 136 cases after five years' observation. Br Med J (1961) 2(5261):1165–72. doi: 10.1136/bmj.2.5261.1165 PMC1970202 14497750

[30] Adegunsoye A Oldham JM Bonham C Hrusch C Nolan P Klejch W. Prognosticating outcomes in interstitial lung disease by mediastinal lymph node assessment. An observational cohort study with independent validation. Am J Respir Crit Care Med (2019) 199(6):747–59. doi: 10.1164/rccm.201804-0761OC PMC6423102 30216085

[31] Ziegenhagen MW Rothe ME Schlaak M Muller-Quernheim J. Bronchoalveolar and serological parameters reflecting the severity of sarcoidosis. Eur Respir J (2003) 21(3):407–13. doi: 10.1183/09031936.03.00010403 12661993

[32] Miyoshi S Hamada H Kadowaki T Hamaguchi N Ito R Irifune K. Comparative evaluation of serum markers in pulmonary sarcoidosis. Chest (2010) 137(6):1391–7. doi: 10.1378/chest.09-1975 20081103

[33] Verwoerd A Hijdra D Vorselaars AD Crommelin HA van Moorsel CH Grutters JC. Infliximab therapy balances regulatory T cells, tumour necrosis factor receptor 2 (TNFR2) expression and soluble TNFR2 in sarcoidosis. Clin Exp Immunol (2016) 185(2):263–70. doi: 10.1111/cei.12808 PMC4955009 27158798

[34] Chilosi M Menestrina F Capelli P Montagna L Lestani M Pizzolo G. Immunohistochemical analysis of sarcoid granulomas. Evaluation of Ki67+ and interleukin-1+ cells. Am J Pathol (1988) 131(2):191–8. PMC1880590 3282443

[35] Ly NTM Ueda-Hayakawa I Nguyen CTH Okamoto H. Exploring the imbalance of circulating follicular helper CD4(+) T cells in sarcoidosis patients. J Dermatol Sci (2020) 97(3):216–24. doi: 10.1016/j.jdermsci.2020.02.002 32063460

