# Supplementary material for: FOXP3+ regulatory T cells are associated with the severity and prognosis of sarcoidosis
Source: Front Immunol. 2023 Dec 20;14:1301991. doi: 10.3389/fimmu.2023.1301991 (PMC10761433; doi:10.3389/fimmu.2023.1301991)
Supplement: Supplementary file 1 [file DataSheet_1.docx]

Supplementary Material

FOXP3+ regulatory T cells are associated with the severity and prognosis of sarcoidosis

Karen C Patterson, Wallace T Miller, Wayne W Hancock and Tatiana Akimova*

*** Correspondence:** Corresponding Author: Tatiana Akimova ttankmail@gmail.com

# Supplementary materials and methods

## Statistical methods.

Evaluation of sample size to study. We used preliminary data of healthy donors Treg suppressive function, evaluated in our laboratory using the same method as we applied in the current study. We hypothesized to observe critical (1/3 or more) impairment of Treg function in subjects with sarcoidosis. T-test for the difference between two independent means of donors Treg function equal to 50 units area under standardized suppressive curve (AUC), and of sarcoid Treg equal to 33 units AUC, with a standard deviation of 20 units, resulted in an effect size of 0.85 (G*Power 3.1.9.4). We set power at 0.8 and significance level α =0.05, along with an allocation ratio of sarcoidosis/donors =2, and determined that we needed to enroll 27 sarcoidosis patients and 13 donors.

Adjustment for FOXP3+ Treg purity used in suppression assay. Patient Tregs had the same mean FOXP3+ purity after CD4^+^CD25^+^ isolation as healthy donor Tregs (Suppl Figure 1F), but demonstrated marked individual variability, which prompted us to adjust the Treg suppression assay results for purity of isolated Tregs, using the same approach we described previously (1).

In the current study, we ran our experiments with four Treg samples, two from sarcoidosis patients and two from healthy donors. Prior to the suppression assays, Tregs were diluted with CD4^+^CD25^-^FOXP3^-^ autologous T cells (Teffs) mimicking their decreased FOXP3^+^ purity, in concentrations of 100 to 30% of originally isolated Tregs. Following this, we ran Treg suppression assays with diluted and non-diluted Tregs from the same sample, and calculated their suppression function as AUC, as previously described (2), separately for CD4^+^ and for CD8^+^ responder T cells. Cryopreserved aliquots of the same Tregs were evaluated by flow cytometry for FOXP3 expression, and the resulted “true” FOXP3 purities of Tregs were calculated for each diluted Treg sample, i.e. if 100% of the (non-diluted) Treg sample showed 80% of FOXP3^+^ expression, then a 50% dilution of that sample had 40% “true” FOXP3^+^ Treg. For each sample, we counted the ratios of the diluted sample AUCs to undiluted sample AUCs. For example, 100% undiluted Tregs had an AUC of 39.87 for CD4^+^ responders, while Tregs diluted to 60% of isolated showed suppression with an AUC of 23.99, and 40% pure Tregs had an AUC of 16.79. As a result, the AUC ratio for 60% Treg dilution was 0.6, and the AUC ratio for 40% pure Treg was 0.42. The AUC ratios of all dilutions of all four tested Treg samples were plotted (GraphPad Prism), where “true” FOXP3 expression was represented on the X axis, and corresponding AUC ratios were plotted on the Y axis. We ran linear regression assays to evaluate how Treg suppressive function changes when FOXP3^+^ purity is decreased, and to evaluate if sarcoidosis Tregs differ from healthy donor counterparts for the same correlation. Despite some individual (or assay related) variabilities, all samples could be described with the same regression analysis equation, one for CD4^+^ and another one for CD8^+^ responder cells (Suppl Figure. S4 A-B).

For the next step, we used the derived equations to predict AUCs for two new Treg samples, one from a sarcoidosis subject and one from a healthy donor. These Tregs were diluted with their Teffs as described above, and their AUCs and AUC ratios were calculated as above, but then compared with predicted AUCs using the corresponding equations for CD4^+^ and CD8^+^ responder cells. Predicted and observed AUCs showed very high concordance (Suppl Figure S4C), which let us to conclude that our adjustment for FOXP3^+^ purity is a valuable tool to control for the effect of possible artifacts in the results of suppression assays that can be caused by individual variabilities of FOXP3^+^ purity of isolated Tregs. For the final step, the AUCs of all isolated donor and sarcoidosis Tregs were re-calculated according to these resulted equations (Suppl. Figure S4 A-C) according to their FOXP3^+^ purities.

Regression models. We performed univariant analyses of all clinical and immunological variables, and further used those with an alpha levels <0.15 for difference regarding need for therapy at 1 year of follow-up.

For regression models, we applied K-Fold cross validation. Typically, k is chosen as 5 or 10, as these values have been shown empirically to yield test error rate estimates that suffer neither from excessively high bias nor from very high variance. Another adopted choice for small datasets is to select the K that gives the testing set with the size about 15% of total dataset. 5 observations per group (16.7% of total ) results with K=6. Subjects were randomly divided into six subsets, and logistic models were run six times, keeping subjects from one of these subsets aside as validation set. The best models were chosen for each run according to best fit and predictive parameters using training sets, and then were tested on validation sets using patients from the corresponding group.

None of the candidate markers were successful as a single variable in regression models, failing to correctly classify patients who needed therapy. As Treg suppressive function had a strong negative correlation with inflammatory markers in plasma and with Ki-67^+^ expression, and as inflammatory markers along with Ki-67 also correlated with each other (Table 2), attempts to combine two and more predictors resulted in a collinearity problem. We therefore combined data using a scoring system with summary “inflammatory scores”, ranging 0 to 8. Six subjects had missing data for one or two markers included in the summary inflammatory scores, in those cases, their missing values were counted as “normal” (i.e. imputed score values of 0).

Probit models outperformed logistic regression models, and the best predictors in both types of models were DLCO (% of predicted) and the inflammatory score. Those predictors of treatment need correctly classified 90% of cases in the training set and 90% of cases in the validation set, with reasonably good overall significance, Likelihood Ratio Chi-Square χ^2^(2) = 10.96, p = 0.004. Data are presented for probit regression models with (Table S5) and without (Table S6) Treg suppressive function as a predictor, and the resulting statistic are shown as medians for all 6 models and interquartile ranges (IQR).

# Supplementary Figures

**
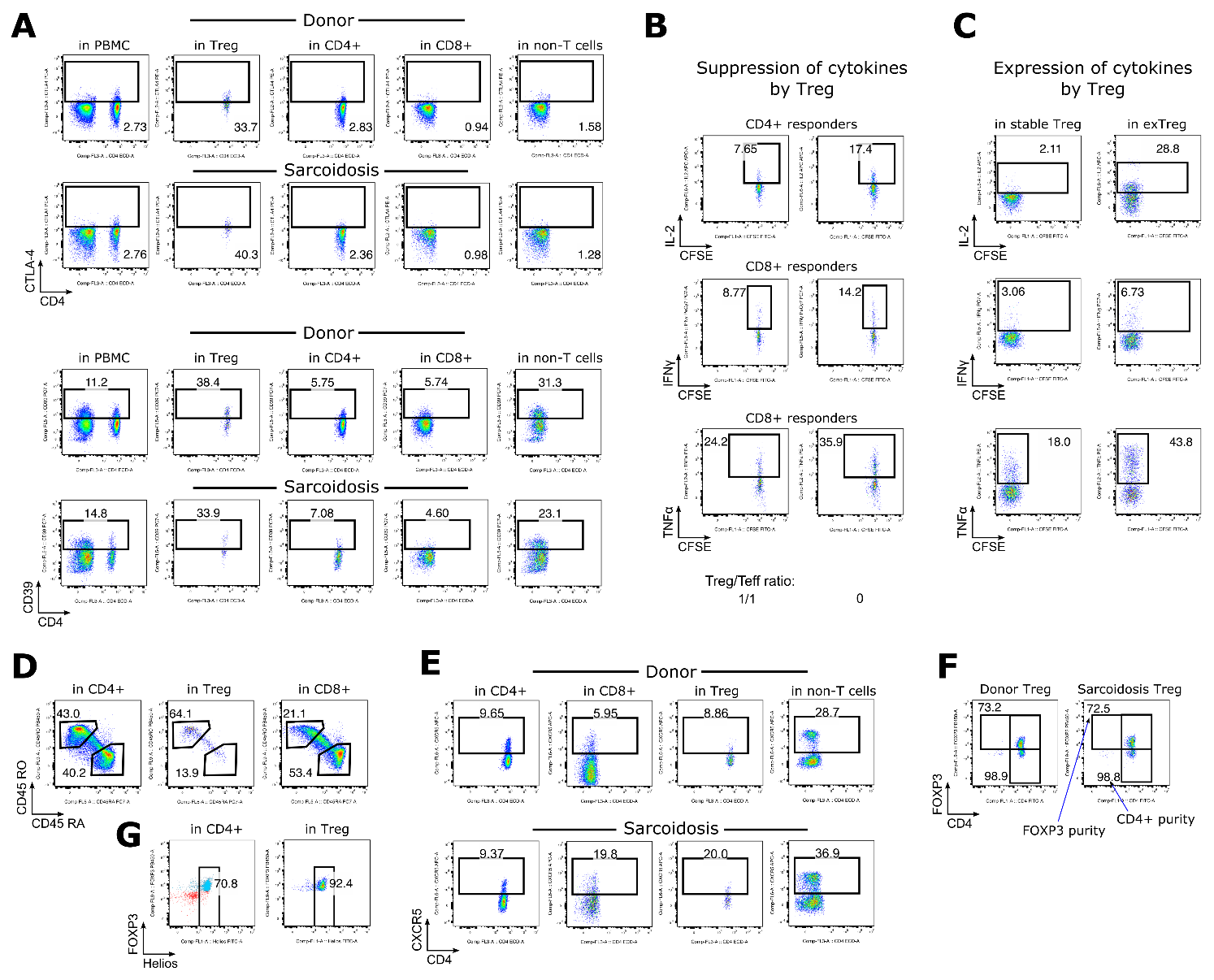
**

**Figure S1. A,** Representative examples and gating strategies for CTLA-4 (top) and CD39 (bottom) expression in different PBMC subsets. **B**, representative examples and gating strategies for the evaluation of cytokine expression, controlled by Tregs, in healthy donors PBMCs. **C**, representative examples and gating strategies for the evaluation of cytokine expression by stable Tregs and exTregs. **D**, gating strategy for the evaluation of CD45RA^+^ and CD45RO^+^ expression in different subsets of T cells. **E**, gating strategy and representative examples of CXCR5 expression in different PBMC subsets. **F**, evaluation of FOXP3^+^ expression in aliquots of isolated Tregs, representative examples. **G**, gating strategy for evaluation of Helios expression in isolated Tregs.


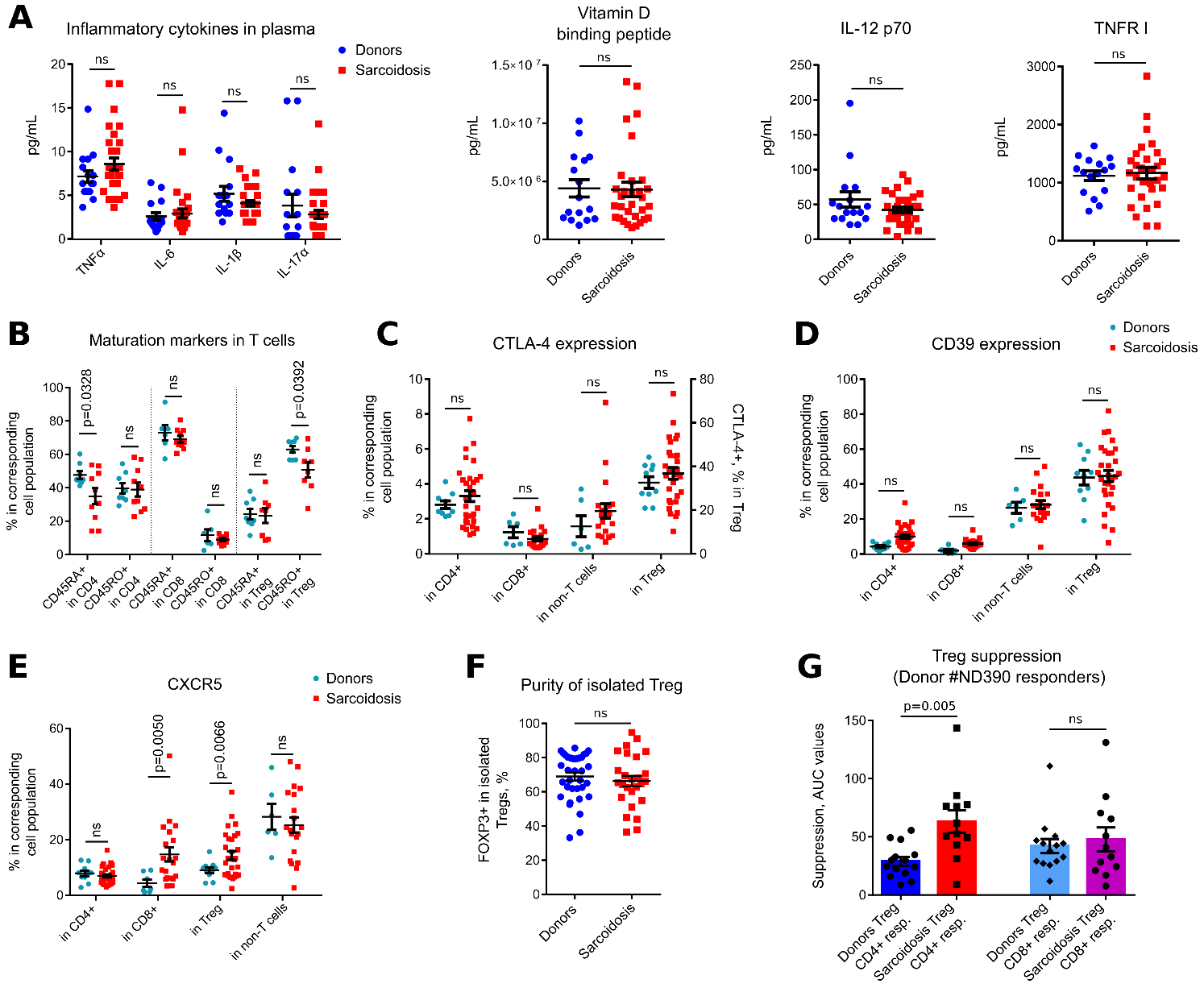


**Figure S2. A,** Plasma levels of TNFα, IL-6, Il-1β, IL17α, Vitamin D binding peptide, IL-12p70 and TNFR I by Luminex assay are displayed, with testing of 29 sarcoidosis patients and 16 donors, matched by age, gender, race and smoking history (Table S2). More Luminex data are presented at Figure 1A. **B**, Expression levels of maturation markers CD45RA and CD45RO evaluated in CD4^+^ and CD8^+^ T cells and in CD4^+^FOXP3^+^ Tregs by flow cytometry. The number of samples for each group (median, IQR): donors: 8, 6-8, sarcoidosis: 10, 8-10. **C-E**, Expression levels of CTLA-4 (**C**), CD39 (**D**) and CXCR5 (**E**) were evaluated in CD4^+^ and CD8^+^ T cells, in CD4^+^FOXP3^+^ Tregs and in CD4^-^CD8^-^ non-T cells by flow cytometry. The number of samples (median, IQR): donors: 8.5, 6.25-10, sarcoidosis 25, 20-30. Gating strategies and representative data for flow cytometry are shown in Figure S1. **F**, FOXP3^+^ expression in CD4^+^CD25^+^ isolated Treg. **G**, suppressive function was evaluated in 14 healthy donors and 12 sarcoidosis patients, using aliquots of responders from the second donor, as described in Methods. Statistics of Treg function adjusted for FOXP3^+^ Treg purity after isolation is demonstrated. More data of Treg suppressive function (with the first donor’s responder cells) are presented in Figure 2A.

**A** (except for TNFR I) and **C-D, F** - Mann-Whitney test; **A** (TNFR I) and **E**, - unpaired t test with Welch's correction; **B** - unpaired T test; **G** - ANOVA + Holm-Sidak test.


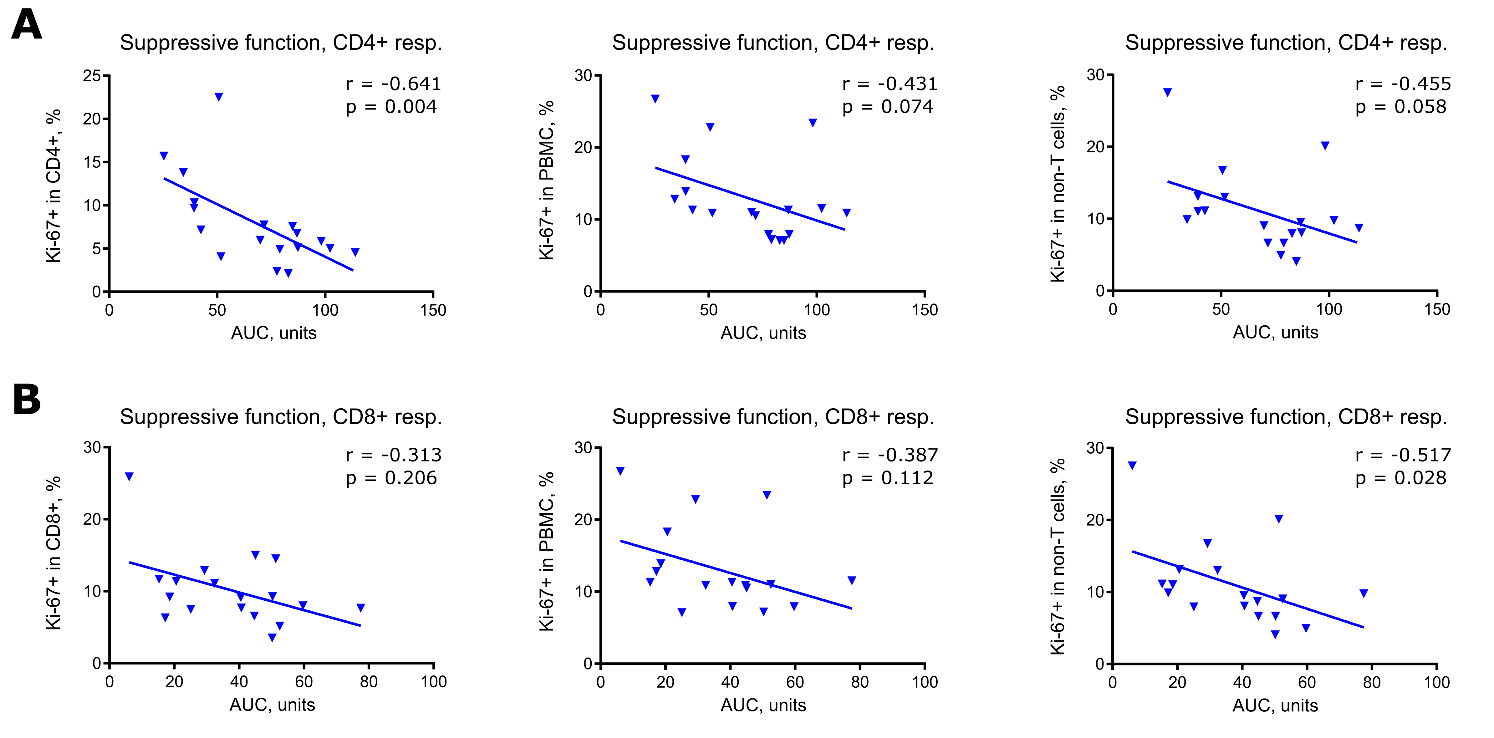


**Figure S3.**

**A-B**, Treg suppressive function in sarcoidosis, calculated for CD4^+^ (**A**) and CD8^+^ (**B**) T cell responders, was inversely correlated with Ki-67 expression in T cells, PBMCs, and in CD4^-^CD8^-^ non-T cells, n = 18. Spearman’s test


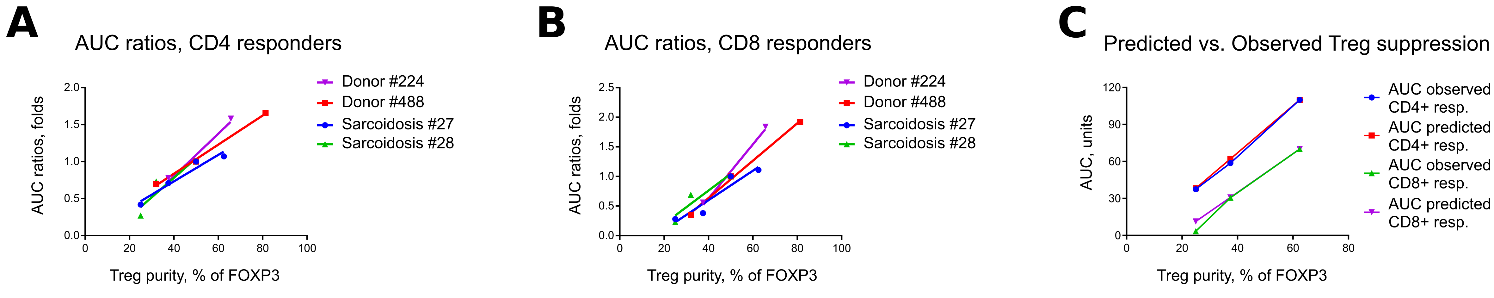


**Figure S4.** **A-C**, - Treg suppressive function was adjusted according to FOXP3^+^ purity of isolated Tregs, as described in Supplementary methods. Ratios of AUCs of 4 Treg samples, diluted with autologous CD4^+^CD25^-^FOXP3^-^Teffs, with concentrations from 100% (no Teffs added) to 30% used. Treg purity was determined by FOXP3^+^ presence in tested Tregs, and is shown at x axes, with the ratios of Treg suppression function to samples with ⁓50% FOXP3^+^ expression shown on the y axes. Linear regression equations describe the loss of Treg suppressive function for CD4^+^ (**A**) and CD8^+^ (**B**) responders. For CD4^+^ responders, the resulted equation is Y = 0.02235*X - 0.1121, goodness of fit R square = 0.9142, Sy.x = 0.1202. For CD8^+^ responders, the resulted equation is Y = 0.03088*X - 0.5477, goodness of fit R square = 0.9063, Sy.x = 0.1742. **C**, a sarcoidosis Treg sample (subject #27) was treated as in **(A, B**), and the resulting AUC ratios were compared with the predicted AUC ratios (using equations **A, B**) for all 3 dilutions of Tregs.

# Supplementary Tables

## Table S1. Immune markers, evaluated in sarcoidosis patients

| **#** | **Markers** | **The reason to choose** | **References** |
| --- | --- | --- | --- |
| Plasma markers, by Luminex* | | | |
| 1 | sCD25/IL-2Ra | - was reported to be increased in sarcoidosis;  - correlates with disease activity  - no correlations with disease activity | (3, 4)  (3-6)  (7, 8) |
| 2 | CXCL10 | - was reported to be increased in sarcoidosis, and to correlate with disease progression | (7, 9) |
| 3 | IFN-γ* | - was reported to be increased in sarcoidosis (serum) | (10, 11) |
| 4 | IL-1β | - was reported to be increased in BALF** in sarcoidosis | (12) |
| 5 | IL-2* | - IL-2 therapy in patients with HIV and cancer was reported to trigger sarcoidosis development or worsen pre-existing sarcoidosis | (13, 14) |
| 6 | IL-6 | - was shown to be increased in systemic sarcoidosis | (15, 16) |
| 7 | IL-10* | - was reported to be increased in sarcoidosis BALF | (17) |
| 8 | IL-12p70 | - was reported to be increased in sarcoidosis BAL cells upon LPS stimulation;  - was shown to be decreased in sarcoidosis plasma | (18)  (19) |
| 9 | IL-17a | - was reported to be increased in sarcoidosis plasma | (20) |
| 10 | IL-18 | - was reported to be increased in sarcoidosis plasma;  - was reported to be increased in BALF cells upon LPS stimulation;  - correlated with disease activity and progression | (11, 21)  (18)  (21) |
| 11 | TNF-α | - was reported to be increased in sarcoidosis | (20) |
| 12,  13 | TNFR I  TNFR II | -were reported to be increased in sarcoidosis and were correlated with disease activity and advanced radiological features;  - TNFR II levels were not increased in sarcoidosis, but were higher in patients who responded to infliximab therapy;  - Treg were shown to shed a large amount of TNFR II | (22-24)  (25)  (26) |
| 14 | Vitamin D  BP*** | -Vitamin D binding peptide was found to be increased in BALF sarcoidosis exosomes | (27) |
| Cellular markers, by flow cytometry | | | |
| 15 | CD4+FOXP3+ | - FOXP3+Treg cells are important regulators of immune response and important in the pathogenesis of multiple diseases, and have been shown to be increased in sarcoidosis blood;  - increased blood Treg numbers were associated with worse outcome;  - increased blood Treg numbers had no clinical associations;  - data of blood Treg function and its association with clinical data are contradictory | (15, 28-31)  (30, 32)  (28)  (29-32) |
| 16 | CD4+CXCR5  CD8+CXCR5+  CD4+FOXP3+  CXCR5+ | - a marker of human CD4+ Tfh subset in the blood, important for germinal center reactions;  - CD4+CXCR5+ was reported to be decreased in sarcoidosis patients;  - CXCR5+ expression was higher in CD45RA-CCR7+CD4+ cells in sarcoidosis, although % of parenting CD45RA-CCR7+ populations were not reported;  - were recently suggested to be important for autoimmune and infection diseases, and were identified within germinal centers;  - no data for sarcoidosis;  - are follicular regulatory T cells (Tfr), controlling germinal center reactions;  - no data for sarcoidosis | (16, 33)  (20)  (34)  (35)  (36) |
| 17 | CD4+FOXP3+  CTLA4+ | - CTLA-4 expression was reported to be decreased in activated Treg cells and T cells from sarcoidosis mediastinal lymph nodes and BALF, although CTLA-4 MFI reported rather then % of positive cells;  - CTLA-4 was reported to be increased in sarcoidosis Treg | (30)  (37) |
| 18 | CD45RA/RO | - CD45RA/RO T cell and Treg subsets were reported to be disturbed in sarcoidosis patients | (28, 37, 38) |
| 19 | Ki-67 | - Ki-67 was reported to be increased in memory T cells from lymph nodes of sarcoidosis patients  - was evaluated in granulomas and associated with disease activity;  - Ki-67 in sarcoidosis blood Treg was reported to be the same as in control Treg | (39)  (40)  (28) |
| 20 | CD4+FOXP3+  CD39 | - CD39 was reported to be increased in sarcoidosis Treg | (41) |
| 21 | Helios | - Helios expression is a marker of T cell and Treg activation and proliferation, however, it has been never reported in sarcoidosis | (42) |

*IL-2, IL-10 and IFN-γ levels were not detectable in most samples by Luminex and were not included in our results.

**BALF = bronchoalveolar lavage fluid

***BP = binding peptide

**Table S2. Age, gender, race, and smoking history of plasma donors**

| **ID** | **Age, years** | **Gender** | **Race** | **Smoking status** |
| --- | --- | --- | --- | --- |
| AD2 | 55 | Male | White | No |
| AD3 | 67 | Male | White | Yes |
| AD4 | 57 | Female | White | No |
| AD5 | 62 | Female | White | No |
| AD6 | 61 | Female | White | No |
| AD7 | 55 | Female | White | Yes |
| AD9 | 57 | Male | Black | Yes |
| AD10 | 56 | Male | Black | Yes |
| AD11 | 34 | Male | White | Yes |
| AD12 | 40 | Male | White | Yes |
| AD13 | 42 | Male | White | Yes |
| AD14 | 46 | Male | White | No |
| AD15 | 31 | Male | Black | No |
| AD16 | 31 | Female | White | Yes |
| AD17 | 33 | Female | White | Yes |
| AD18 | 32 | Female | Black | No |
| **Total/average** | **47.44+/-3.17 years** | **56% males** | **25% black** | **56% smokers*** |

*Smoking status, although not ideally matched, does not significantly differ vs. sarcoidosis cohort, p = 0.36 by Fisher’s exact test.

**Table S3. Flow cytometry antibodies and reagents**

| **Flow cytometry** | | | | |
| --- | --- | --- | --- | --- |
| **#** | **Name** | **Clone/catalog #** | **Color/Type/PRID** | **Manufacturer** |
| 1 | CFSE | cat# C1157 | 492/517 nm | Life Technologies |
| 2 | Live/dead fixable | cat# L34957 | 405/525 nm | Life Technologies |
| 3 | Zombie Yellow fixable | cat# 423103 | 405/572 nm | Biolegend |
| 4 | Zombie Aqua fixable | cat#423102 | 405/516 | Biolegend |
| 5 | Ghost Dye, violet 510 | cat#13-0870-T100 | 405/510 | Tonbo Bioscience |
| 6 | CD4 | RPA-T4 | PE-CF594 | BD Biosciences |
| 7 | CD4 | SK3 | APC (RRID:AB_2028488), APC-Cy7 (RRID:AB_2028483), PE (RRID:AB_1937246), FITC (RRID:AB_2616621), PerCP/Cy5.5 (RRID:AB_2820223), AF700 (RRID:AB_2563150) | Biolegend |
| 8 | CD8 | HIT8a | PerCP/Cy5.5, FITC, APC-Cy7 | Biolegend |
| 9 | CD8a | RPA-T8 | PE-CF594 (RRID:AB_2869914) | BD Biosciences |
| 10 | CD39 | eBioA1 (A1) | Pe-Cy7 (RRID:AB_1582280) | eBioscience |
| 11 | CD45 | HI30 | FITC (RRID:AB_314394), AF700 (RRID:AB_493761), redFluor™ 710 (RRID:AB_2621990) | Biolegend, Tonbo Biosciences |
| 12 | CD45RA | HI100 | Pe-Cy7 (RRID:AB_10708879) | Biolegend |
| 13 | CD45RO | UCHL1 | BV-421 (RRID:AB_2563817) | Biolegend |
| 14 | CD152 (CTLA-4) | BNI3 | PE (RRID:AB_396628) | BD Biosciences |
| 15 | CD185 (CXCR5) | J252D4 | AF647 (RRID:AB_2561815) | Biolegend |
| 16 | FOXP3 | PCH101 | AF647, eFluor 450 (RRID:AB_1834364) | eBioscience |
| 17 | Helios | 22F6 | FITC (RRID:AB_10662745) | Biolegend |
| 18 | Ki-67 | cat#561284 | PerCP-Cy5.5 (RRID:AB_10611574) | BD Biosciences |
| 19 | Ki-67 | cat#46-5699-42 | PerCP-eFluor® 710 (RRID:AB_10804653) | eBioscience |
| 20 | IL-2 | MQ1-17H12 | APC (RRID:AB_315097) | Biolegend |
| 21 | IFNγ | B27 | Pe-Cy7 (RRID:AB_2123321) | Biolegend |
| 22 | TNFα | MAb11 | PE (RRID:AB_315261) | Biolegend |
| 23 | Monocytes blocking | cat#426102 | True-Stain Monocyte Blocker | Biolegend |
| 24 | Fc blocking | cat#422302 | Human TruStain FcX™ (RRID:AB_2818986) | Biolegend |
| 25 | Cytokines assay | cat#420701 | Monensin Solution | Biolegend |
| 26 | Cytokines assay | cat#420601 | Brefeldin A Solution | Biolegend |
| 27 | Fix/perm | cat#562574 | Transcription Factor Buffer Set | BD Biosciences |
| **Stimulation** | | | | |
| 1 | CD3 mAbs-coated beads | OKT3  and  Dynabeads | MACS GMP pure (RRID:AB_2904535) and M-450 Tosylactivated beads | Miltenyi Biotec and  Life Technologies |
| 2 | PMA | cat# P8139-1MG | Phorbol 12-myristate 13-acetate | Sigma-Aldrich Co |
| 3 | Ionomycin | cat# I0634-1MG | Ionomycin calcium salt | Sigma-Aldrich Co |

**Table S4. Correlation matrix of inflammatory markers in plasma of healthy donors**

| **Plasma markers** | | IL18 in plasma | IL1β in plasma | TNFα in plasma | IL-6 in plasma | CXCL10 in plasma | IL-17a in plasma | sCD25/IL2Ra in plasma | IL12p70 in plasma | TNFR II in plasma | TNFR I in plasma |
| --- | --- | --- | --- | --- | --- | --- | --- | --- | --- | --- | --- |
| IL18 in plasma | r | 1.000 | .080 | .325 | .337 | -.060 | .144 | .330 | .372 | .277 | .149 |
|  | Sig. | . | .768 | .219 | .202 | .824 | .594 | .212 | .156 | .300 | .583 |
|  | N | 16 | 16 | 16 | 16 | 16 | 16 | 16 | 16 | 16 | 16 |
| IL1β in plasma | r | .080 | 1.000 | .540* | .563* | -.736** | .635** | -.165 | .717** | -.423 | .015 |
|  | Sig. | .768 | . | .031 | .023 | .001 | .008 | .542 | .002 | .103 | .956 |
|  | N | 16 | 16 | 16 | 16 | 16 | 16 | 16 | 16 | 16 | 16 |
| TNFα in plasma | r | .325 | .540* | 1.000 | .566* | -.097 | .616* | .428 | .666** | .097 | .442 |
|  | Sig. | .219 | .031 | . | .022 | .722 | .011 | .098 | .005 | .722 | .086 |
|  | N | 16 | 16 | 16 | 16 | 16 | 16 | 16 | 16 | 16 | 16 |
| IL-6 in plasma | r | .337 | .563* | .566* | 1.000 | -.238 | .753** | -.029 | .531* | -.049 | .201 |
|  | Sig. | .202 | .023 | .022 | . | .375 | .001 | .916 | .034 | .858 | .456 |
|  | N | 16 | 16 | 16 | 16 | 16 | 16 | 16 | 16 | 16 | 16 |
| CXCL10 in plasma | r | -.060 | -.736** | -.097 | -.238 | 1.000 | -.337 | .284 | -.603* | .656** | .482 |
|  | Sig. | .824 | .001 | .722 | .375 | . | .201 | .286 | .013 | .006 | .058 |
|  | N | 16 | 16 | 16 | 16 | 16 | 16 | 16 | 16 | 16 | 16 |
| IL-17a in plasma | r | .144 | .635** | .616* | .753** | -.337 | 1.000 | -.276 | .608* | -.243 | .055 |
|  | Sig. | .594 | .008 | .011 | .001 | .201 | . | .301 | .012 | .364 | .838 |
|  | N | 16 | 16 | 16 | 16 | 16 | 16 | 16 | 16 | 16 | 16 |
| sCD25/IL2Ra in plasma | r | .330 | -.165 | .428 | -.029 | .284 | -.276 | 1.000 | .034 | .475 | .590* |
|  | Sig. | .212 | .542 | .098 | .916 | .286 | .301 | . | .900 | .063 | .016 |
|  | N | 16 | 16 | 16 | 16 | 16 | 16 | 16 | 16 | 16 | 16 |
| IL12p70 in plasma | r | .372 | .717** | .666** | .531* | -.603* | .608* | .034 | 1.000 | -.246 | .009 |
|  | Sig. | .156 | .002 | .005 | .034 | .013 | .012 | .900 | . | .358 | .974 |
|  | N | 16 | 16 | 16 | 16 | 16 | 16 | 16 | 16 | 16 | 16 |
| TNFR II in plasma | r | .277 | -.423 | .097 | -.049 | .656** | -.243 | .475 | -.246 | 1.000 | .762** |
|  | Sig. | .300 | .103 | .722 | .858 | .006 | .364 | .063 | .358 | . | .001 |
|  | N | 16 | 16 | 16 | 16 | 16 | 16 | 16 | 16 | 16 | 16 |
| TNFR I in plasma | r | .149 | .015 | .442 | .201 | .482 | .055 | .590* | .009 | .762** | 1.000 |
|  | Sig. | .583 | .956 | .086 | .456 | .058 | .838 | .016 | .974 | .001 | . |
|  | N | 16 | 16 | 16 | 16 | 16 | 16 | 16 | 16 | 16 | 16 |

Positive significant correlations (Spearman) highlighted at yellow; negative significant correlations highlighted at blue, where significance is adjusted by the Benjamini–Hochberg multiple comparison procedure(43) at p<0.0067, 2-tailed.

## Table S5. Probit regression model with Treg function

| **Omnibus Test** | | | **Test of Model Effects (Type III)** | | | |
| --- | --- | --- | --- | --- | --- | --- |
|  |  |  | **Source** | **Likelihood Ratio Chi-Square, (IQR)** | **df** | **Sig., (IQR)** |
| **Likelihood Ratio Chi-Square, (IQR)** | **df** | **Sig., (IQR)** | DLCO % predicted | 9.76,  (9.43-10.80) | 1 | 0.002,  (0.001-0.002) |
| 10.96, (9.98-12.00) | 2 | 0.004,  (0.002-0.007) | Inflam. score: Treg function, TNFα, CXCL10, sCD25, TNFR I and TNFR II, Ki-67 | 7.17,  (6.69-7.76) | 1 | 0.007,  (0.005-0.01) |

**Parameter Estimates**

| **Parameter** | **B, (IQR)** | **Std. Error, (IQR)** | **Hypothesis Test** | | | **95% Profile Likelihood Confidence Interval for Exp(B)** | | |
| --- | --- | --- | --- | --- | --- | --- | --- | --- |
|  |  |  | **Wald Chi-Square** | **df** | **Sig., (IQR)** | **Exp(B), (IQR)** | **Lower, (IQR)** | **Upper, (IQR)** |
| DLCO % predicted | -0.045, (- 0.046- - 0.045) | 0.020,  (0.020-0.021) | 4.87,  (4.26-5.32) | 1 | 0.028,  (0.021-0.039) | 0.956,  (0.955– 0.956) | 0.914,  (0.907-0.914) | 0.987,  (0.986-0.988) |
| Inflam. score: Treg function, TNFα, CXCL10, sCD25, TNFR1 and TNFR2, Ki-67 | 0.569, (0.562-0.578) | 0.272,  (0.263-0.318) | 4.25,  (3.17-4.77) | 1 | 0.040,  (0.029-0.078) | 1.767,  (1.755-1.783) | 1.138,  (1.117-1.151) | 3.253,  (3.214-3.881) |

**Classification Table (validation set)**

| **Observed** | | **Predicted** | | |
| --- | --- | --- | --- | --- |
|  |  | **On therapy at 1 year follow up:** | | **Percentage Correct, %** |
|  |  | **No** | **Yes** |  |
| **On therapy at 1 year follow up:** | **No** | 13 | 1 | 92.9 |
|  | **Yes** | 1 | 5 | 83.3 |
| **Overall Percentage** | |  |  | 90 |

**Classification Table (validation set, treatment-naïve patients only)**

| **Observed** | | **Predicted** | | |
| --- | --- | --- | --- | --- |
|  |  | **On therapy at 1 year follow up:** | | **Percentage Correct, %** |
|  |  | **No** | **Yes** |  |
| **On therapy at 1 year follow up:** | **No** | 9 | 1 | 90.0 |
|  | **Yes** | 1 | 3 | 75.0 |
| **Overall Percentage** | |  |  | 85.7 |

**Classification Table (training set)**

| **Observed** | | **Predicted** | | |
| --- | --- | --- | --- | --- |
|  |  | **On therapy at 1 year follow up:** | | **Percentage Correct, %** |
|  |  | **No** | **Yes** |  |
| **On therapy at 1 year follow up:** | **No** | 65 | 5 | 92.9 |
|  | **Yes** | 5 | 25 | 83.3 |
| **Overall Percentage** | |  |  | 90.0 |

Dependent variable: on therapy at 1 year follow up. Predictors: inflammatory score and DLCO % predicted. Data for all 6 models reported as Median and IQR (interquartile range). For classification tables, summary of all models shown.

## Table S6. Probit regression model without Treg function

| **Omnibus Test** | | | **Test of Model Effects (Type III)** | | | |
| --- | --- | --- | --- | --- | --- | --- |
|  |  |  | **Source** | **Likelihood Ratio Chi-Square, (IQR)** | **df** | **Sig., (IQR)** |
| **Likelihood Ratio Chi-Square, (IQR)** | **df** | **Sig., (IQR)** | DLCO % predicted | 6.720,  (6.284-7.194) | 1 | 0.010,  (0.007-0.012) |
| 8.242, (7.533-9.128) | 2 | 0.016,  (0.011-0.023) | Inflam. score: TNFα, CXCL10, sCD25, TNFR I and TNFR II, Ki-67 | 4.447,  (3.757-4.979) | 1 | 0.035,  (0.026-0.053) |

**Parameter Estimates**

| **Parameter** | **B, (IQR)** | **Std. Error, (IQR)** | **Hypothesis Test** | | | **95% Profile Likelihood Confidence Interval for Exp(B)** | | |
| --- | --- | --- | --- | --- | --- | --- | --- | --- |
|  |  |  | **Wald Chi-Square** | **df** | **Sig., (IQR)** | **Exp(B), (IQR)** | **Lower, (IQR)** | **Upper, (IQR)** |
| DLCO % predicted | -0.037, (- 0.037- - 0.035) | 0.018,  (0.018-0.019) | 3.785,  (3.631-3.929) | 1 | 0.052,  (0.048-0.057) | 0.964,  (0.963– 0.966) | 0.926,  (0.923-0.928) | 0.993,  (0.992-0.994) |
| Inflam. score: TNFα, CXCL10, sCD25, TNFR1 and TNFR2, Ki-67 | 0.535, (0.482-0.548) | 0.295,  (0.292-0.313) | 3.165,  (2.612-3.459) | 1 | 0.076,  (0.063-0.107) | 1.707,  (1.621-1.731) | 1.032,  (0.996-1.055) | 3.190,  (3.071-3.489) |

**Classification Table (validation set)**

| **Observed** | | **Predicted** | | |
| --- | --- | --- | --- | --- |
|  |  | **On therapy at 1 year follow up:** | | **Percentage Correct, %** |
|  |  | **No** | **Yes** |  |
| **On therapy at 1 year follow up:** | **No** | 13 | 1 | 92.9 |
|  | **Yes** | 3 | 3 | 50.0 |
| **Overall Percentage** | |  |  | 80 |

**Classification Table (validation set, treatment-naïve patients only)**

| **Observed** | | **Predicted** | | |
| --- | --- | --- | --- | --- |
|  |  | **On therapy at 1 year follow up:** | | **Percentage Correct, %** |
|  |  | **No** | **Yes** |  |
| **On therapy at 1 year follow up:** | **No** | 10 | 0 | 100.0 |
|  | **Yes** | 3 | 1 | 25.0 |
| **Overall Percentage** | |  |  | 78.6 |

**Classification Table (training set)**

| **Observed** | | **Predicted** | | |
| --- | --- | --- | --- | --- |
|  |  | **On therapy at 1 year follow up:** | | **Percentage Correct, %** |
|  |  | **No** | **Yes** |  |
| **On therapy at 1 year follow up:** | **No** | 66 | 4 | 94.3 |
|  | **Yes** | 12 | 18 | 60.0 |
| **Overall Percentage** | |  |  | 84.0 |

Dependent variable: on therapy at 1 year follow up. Predictors: inflammatory score without Treg function and DLCO % predicted. Data for all 6 models reported as Median and IQR (interquartile range). For classification tables, summary of all models shown.

# Supplementary references

1. Akimova T, Zhang T, Negorev D, Singhal S, Stadanlick J, Rao A, et al. Human lung tumor FOXP3+ Tregs upregulate four "Treg-locking" transcription factors. JCI Insight. 2017;2(16).

2. Akimova T, Levine MH, Beier UH, Hancock WW. Standardization, Evaluation, and Area-Under-Curve Analysis of Human and Murine Treg Suppressive Function. Methods Mol Biol. 2016;1371:43-78.

3. Thi Hong Nguyen C, Kambe N, Kishimoto I, Ueda-Hayakawa I, Okamoto H. Serum soluble interleukin-2 receptor level is more sensitive than angiotensin-converting enzyme or lysozyme for diagnosis of sarcoidosis and may be a marker of multiple organ involvement. J Dermatol. 2017;44(7):789-97.

4. Eurelings LEM, Miedema JR, Dalm V, van Daele PLA, van Hagen PM, van Laar JAM, et al. Sensitivity and specificity of serum soluble interleukin-2 receptor for diagnosing sarcoidosis in a population of patients suspected of sarcoidosis. PLoS One. 2019;14(10):e0223897.

5. Ziegenhagen MW, Rothe ME, Schlaak M, Muller-Quernheim J. Bronchoalveolar and serological parameters reflecting the severity of sarcoidosis. Eur Respir J. 2003;21(3):407-13.

6. Miyoshi S, Hamada H, Kadowaki T, Hamaguchi N, Ito R, Irifune K, et al. Comparative evaluation of serum markers in pulmonary sarcoidosis. Chest. 2010;137(6):1391-7.

7. Su R, Nguyen ML, Agarwal MR, Kirby C, Nguyen CP, Ramstein J, et al. Interferon-inducible chemokines reflect severity and progression in sarcoidosis. Respir Res. 2013;14:121.

8. Grutters JC, Fellrath JM, Mulder L, Janssen R, van den Bosch JM, van Velzen-Blad H. Serum soluble interleukin-2 receptor measurement in patients with sarcoidosis: a clinical evaluation. Chest. 2003;124(1):186-95.

9. Arger NK, Ho ME, Allen IE, Benn BS, Woodruff PG, Koth LL. CXCL9 and CXCL10 are differentially associated with systemic organ involvement and pulmonary disease severity in sarcoidosis. Respiratory Medicine. 2020;161:105822.

10. Shigehara K, Shijubo N, Ohmichi M, Kamiguchi K, Takahashi R, Morita-Ichimura S, et al. Increased circulating interleukin-12 (IL-12) p40 in pulmonary sarcoidosis. Clin Exp Immunol. 2003;132(1):152-7.

11. Shigehara K, Shijubo N, Ohmichi M, Yamada G, Takahashi R, Okamura H, et al. Increased levels of interleukin-18 in patients with pulmonary sarcoidosis. Am J Respir Crit Care Med. 2000;162(5):1979-82.

12. Ringkowski S, Thomas PS, Herbert C. Interleukin-12 family cytokines and sarcoidosis. Front Pharmacol. 2014;5:233.

13. Blanche P, Gombert B, Rollot F, Salmon D, Sicard D. Sarcoidosis in a patient with acquired immunodeficiency syndrome treated with interleukin-2. Clin Infect Dis. 2000;31(6):1493-4.

14. Logan TF, Bensadoun ES. Increased disease activity in a patient with sarcoidosis after high dose interleukin 2 treatment for metastatic renal cancer. Thorax. 2005;60(7):610-1.

15. Celada LJ, Kropski JA, Herazo-Maya JD, Luo W, Creecy A, Abad AT, et al. PD-1 up-regulation on CD4(+) T cells promotes pulmonary fibrosis through STAT3-mediated IL-17A and TGF-beta1 production. Sci Transl Med. 2018;10(460).

16. Chevalier N, Jarrossay D, Ho E, Avery DT, Ma CS, Yu D, et al. CXCR5 expressing human central memory CD4 T cells and their relevance for humoral immune responses. J Immunol. 2011;186(10):5556-68.

17. Barbarin V, Petrek M, Kolek V, Van Snick J, Huaux F, Lison D. Characterization of p40 and IL-10 in the BALF of patients with pulmonary sarcoidosis. J Interferon Cytokine Res. 2003;23(8):449-56.

18. Shigehara K, Shijubo N, Ohmichi M, Takahashi R, Kon S, Okamura H, et al. IL-12 and IL-18 are increased and stimulate IFN-gamma production in sarcoid lungs. J Immunol. 2001;166(1):642-9.

19. Antoniou KM, Tzouvelekis A, Alexandrakis MG, Tsiligianni I, Tzanakis N, Sfiridaki K, et al. Upregulation of Th1 cytokine profile (IL-12, IL-18) in bronchoalveolar lavage fluid in patients with pulmonary sarcoidosis. J Interferon Cytokine Res. 2006;26(6):400-5.

20. Ly NTM, Ueda-Hayakawa I, Nguyen CTH, Okamoto H. Exploring the imbalance of circulating follicular helper CD4(+) T cells in sarcoidosis patients. J Dermatol Sci. 2020;97(3):216-24.

21. Kieszko R, Krawczyk P, Jankowska O, Chocholska S, Krol A, Milanowski J. The clinical significance of interleukin 18 assessment in sarcoidosis patients. Respir Med. 2007;101(4):722-8.

22. Ziegenhagen MW, Fitschen J, Martinet N, Schlaak M, Muller-Quernheim J. Serum level of soluble tumour necrosis factor receptor II (75 kDa) indicates inflammatory activity of sarcoidosis. J Intern Med. 2000;248(1):33-41.

23. Kieszko R, Krawczyk P, Chocholska S, Bojarska-Junak A, Jankowska O, Krol A, et al. Tumor necrosis factor receptors (TNFRs) on T lymphocytes and soluble TNFRs in different clinical courses of sarcoidosis. Respir Med. 2007;101(3):645-54.

24. Nakayama T, Hashimoto S, Amemiya E, Horie T. Elevation of plasma-soluble tumour necrosis factor receptors (TNF-R) in sarcoidosis. Clin Exp Immunol. 1996;104(2):318-24.

25. Verwoerd A, Hijdra D, Vorselaars AD, Crommelin HA, van Moorsel CH, Grutters JC, et al. Infliximab therapy balances regulatory T cells, tumour necrosis factor receptor 2 (TNFR2) expression and soluble TNFR2 in sarcoidosis. Clin Exp Immunol. 2016;185(2):263-70.

26. van Mierlo GJ, Scherer HU, Hameetman M, Morgan ME, Flierman R, Huizinga TW, et al. Cutting edge: TNFR-shedding by CD4+CD25+ regulatory T cells inhibits the induction of inflammatory mediators. J Immunol. 2008;180(5):2747-51.

27. Martinez-Bravo MJ, Wahlund CJ, Qazi KR, Moulder R, Lukic A, Radmark O, et al. Pulmonary sarcoidosis is associated with exosomal vitamin D-binding protein and inflammatory molecules. J Allergy Clin Immunol. 2017;139(4):1186-94.

28. Taflin C, Miyara M, Nochy D, Valeyre D, Naccache JM, Altare F, et al. FoxP3+ regulatory T cells suppress early stages of granuloma formation but have little impact on sarcoidosis lesions. Am J Pathol. 2009;174(2):497-508.

29. Oswald-Richter KA, Richmond BW, Braun NA, Isom J, Abraham S, Taylor TR, et al. Reversal of global CD4+ subset dysfunction is associated with spontaneous clinical resolution of pulmonary sarcoidosis. J Immunol. 2013;190(11):5446-53.

30. Broos CE, van Nimwegen M, In 't Veen JC, Hoogsteden HC, Hendriks RW, van den Blink B, et al. Decreased Cytotoxic T-Lymphocyte Antigen 4 Expression on Regulatory T Cells and Th17 Cells in Sarcoidosis: Double Trouble? Am J Respir Crit Care Med. 2015;192(6):763-5.

31. Rappl G, Pabst S, Riemann D, Schmidt A, Wickenhauser C, Schutte W, et al. Regulatory T cells with reduced repressor capacities are extensively amplified in pulmonary sarcoid lesions and sustain granuloma formation. Clin Immunol. 2011;140(1):71-83.

32. Miyara M, Amoura Z, Parizot C, Badoual C, Dorgham K, Trad S, et al. The immune paradox of sarcoidosis and regulatory T cells. J Exp Med. 2006;203(2):359-70.

33. Morita R, Schmitt N, Bentebibel SE, Ranganathan R, Bourdery L, Zurawski G, et al. Human blood CXCR5(+)CD4(+) T cells are counterparts of T follicular cells and contain specific subsets that differentially support antibody secretion. Immunity. 2011;34(1):108-21.

34. Kudryavtsev I, Serebriakova M, Starshinova A, Zinchenko Y, Basantsova N, Malkova A, et al. Imbalance in B cell and T Follicular Helper Cell Subsets in Pulmonary Sarcoidosis. Sci Rep. 2020;10(1):1059.

35. Valentine KM, Hoyer KK. CXCR5+ CD8 T Cells: Protective or Pathogenic? Front Immunol. 2019;10:1322.

36. Huang Y, Chen Z, Wang H, Ba X, Shen P, Lin W, et al. Follicular regulatory T cells: a novel target for immunotherapy? Clin Transl Immunology. 2020;9(2):e1106.

37. Broos CE, Van Nimwegen M, Kleinjan A, Ten Berge B, Muskens F, In ’T Veen JCCM, et al. Impaired survival of regulatory T cells in pulmonary sarcoidosis. 2015;16(1).

38. Ramstein J, Broos CE, Simpson LJ, Ansel KM, Sun SA, Ho ME, et al. IFN-gamma-Producing T-Helper 17.1 Cells Are Increased in Sarcoidosis and Are More Prevalent than T-Helper Type 1 Cells. Am J Respir Crit Care Med. 2016;193(11):1281-91.

39. Broos CE, Koth LL, van Nimwegen M, In 't Veen J, Paulissen SMJ, van Hamburg JP, et al. Increased T-helper 17.1 cells in sarcoidosis mediastinal lymph nodes. Eur Respir J. 2018;51(3).

40. Chilosi M, Menestrina F, Capelli P, Montagna L, Lestani M, Pizzolo G, et al. Immunohistochemical analysis of sarcoid granulomas. Evaluation of Ki67+ and interleukin-1+ cells. Am J Pathol. 1988;131(2):191-8.

41. Kudryavtsev IV, Lazareva NM, Baranova OP, Golovkin AS, Isakov DV, Serebriakova MK, et al. CD39+ REGULATORY T CELLS IN PULMONARY SARCOIDOSIS AND LOFGREN'S SYNDROME. Medical Immunology (Russia). 2019;21:467-78.

42. Akimova T, Beier UH, Wang L, Levine MH, Hancock WW. Helios expression is a marker of T cell activation and proliferation. PLoS One. 2011;6(8):e24226.

43. Lewis C. Multiple Comparisons. In: Peterson P, Baker E, McGaw B, editors. International Encyclopedia of Education (Third Edition). Oxford: Elsevier; 2010. p. 312-8.
